# Identification of an Enhancer That Increases miR-200b~200a~429 Gene Expression in Breast Cancer Cells

**DOI:** 10.1371/journal.pone.0075517

**Published:** 2013-09-25

**Authors:** Joanne L. Attema, Andrew G. Bert, Yat-Yuen Lim, Natasha Kolesnikoff, David M. Lawrence, Katherine A. Pillman, Eric Smith, Paul A. Drew, Yeesim Khew-Goodall, Frances Shannon, Gregory J. Goodall

**Affiliations:** 1 Centre for Cancer Biology, SA Pathology, Adelaide, South Australia, Australia; 2 Discipline of Medicine, the University of Adelaide, Adelaide, South Australia, Australia; 3 Institute of Biological Sciences, Faculty of Science, University of Malaya, Kuala Lumpur, Malaysia; 4 ACRF Cancer Genomics Facility, Centre for Cancer Biology, Adelaide, South Australia, Australia; 5 Discipline of Surgery, the University of Adelaide, Adelaide, South Australia, Australia; 6 School of Nursing and Midwifery, Flinders University, Bedford Park, South Australia, Australia; 7 Office of Deputy Vice-Chancellor (Research), the University of Canberra, Bruce, Australian Capital Territory, Australia; 8 School of Molecular and Biomedical Science, the University of Adelaide, Adelaide, South Australia, Australia; The University of Arizona, United States of America

## Abstract

The miR-200b~200a~429 gene cluster is a key regulator of EMT and cancer metastasis, however the transcription-based mechanisms controlling its expression during this process are not well understood. We have analyzed the miR-200b~200a~429 locus for epigenetic modifications in breast epithelial and mesenchymal cell lines using chromatin immunoprecipitation assays and DNA methylation analysis. We discovered a novel enhancer located approximately 5.1kb upstream of the miR-200b~200a~429 transcriptional start site. This region was associated with the active enhancer chromatin signature comprising H3K4me1, H3K27ac, RNA polymerase II and CpG dinucleotide hypomethylation. Luciferase reporter assays revealed the upstream enhancer stimulated the transcription of the miR-200b~200a~429 minimal promoter region approximately 27-fold in breast epithelial cells. Furthermore, we found that a region of the enhancer was transcribed, producing a short, GC-rich, mainly nuclear, non-polyadenylated RNA transcript designated *miR-200b eRNA*. Over-expression of *miR-200b eRNA* had little effect on miR-200b~200a~429 promoter activity and its production did not correlate with miR-200b~200a~429 gene expression. While additional investigations of *miR-200b eRNA* function will be necessary, it is possible that *miR-200b eRNA* may be involved in the regulation of miR-200b~200a~429 gene expression and silencing. Taken together, these findings reveal the presence of a novel enhancer, which contributes to miR-200b~200a~429 transcriptional regulation in epithelial cells.

## Introduction

MicroRNAs (miRNAs) are small noncoding RNAs that regulate gene expression programs for a number of critical cellular pathways, including stem cell identity, differentiation, cell division and lineage commitment [1]. There is increasing evidence that miRNAs can act as master regulators of epithelial mesenchymal transition (EMT), an early developmental process that is also linked to tumor cell migration and the establishment of secondary metastases [2]. One such miRNA involved in EMT and cancer metastasis is the miR-200 gene family. The miR-200 family comprises five members (miR-200a, miR-200b, miR-200c, miR-141 and miR-429), clustered and expressed as two separate polycistronic pri-miRNA transcripts, with the miR-200b~200a~429 gene cluster at chromosomal location 1p36 and miR-200c~141 cluster at chromosomal location 12p13 [3]. Numerous laboratories have shown that EMT is induced by the loss of expression of the miR-200 family, which enables maintenance of the epithelial phenotype [4–9]. A double negative feedback-loop between the ZEB1/2 transcription factors and the miR-200 genes regulates the induction of EMT and the reverse process, mesenchymal to epithelial transition (MET) [4,5,7]. Although the promoter regions of the miR-200 genes are well defined [4], less is known about the transcriptional mechanisms controlling the expression of this particular gene family in epithelial cells.

Spatiotemporal control of gene expression is a complex process involving the coordinated actions of transcription factors, chromatin modifying enzymes as well as distinct classes of functional genomic elements including promoters, insulator elements and enhancers. Enhancers are proposed to be the most abundant class of regulatory elements, comprising up to 10% of the human genome [10]. Enhancers have the unique ability to act across long distances and in an orientation independent manner, interacting with factors to enhance transcription [11]. Mechanistically, these elements function by recruiting sequence-specific transcription factors and co-activator complexes and delivering them to distally located promoters throughout the genome. They are generally protected from CpG methylation, creating an accessible chromatin configuration for transcription factor binding and long-range promoter interactions [12,13]. Recent global high throughput technologies, including next generation sequencing chromatin immunoprecipitation assays (ChIP-seq), have advanced the identification and biology of enhancer elements [14,15]. Enhancers can be identified by a H3K4 methylation signature comprising higher amounts of H3K4 monomethylation (H3K4me1) and lower amounts of H3K4 trimethylation (H3K4me3) [16,17]. Additional subclasses of active, intermediate and poised H3K4me1-enriched enhancers have been identified based on their differential co-association with H3K27 acetylation (H3K27ac), H3K9ac, H3K4me2, H3K4me3, H3K27me3, RNA polymerase II (RNAPII), and the histone acetyltransferase, p300 [18–24].

Recent studies indicate that active enhancers produce noncoding RNA transcripts, termed enhancer RNAs (eRNAs) [21,25–28]. These transcripts are typically non-polyadenylated, less than 2000 nucleotides in length, not spliced, and their nuclear localization suggests a role in transcriptional processes [26,29]. It has been proposed that active enhancers may also be promoters regulating noncoding RNA expression in addition to their enhancer function [30,31]. Recent studies propose that eRNAs bind transcriptional co-activators and chromatin modifying complexes, mediate chromatin looping of enhancer elements with promoters *in cis*, and provide a structural scaffold for factors that regulate chromatin and gene expression [25,26,29]. Alternatively, the eRNAs might result from collisions of RNAPII with genomic regions or RNAPII interactions during long distance looping of enhancers to promoters [32]. Nonetheless, the function and biological roles of eRNAs remains to be determined.

Transcription of the primary miR-200b~200a~429 transcript is controlled by a well defined transcriptional start site (TSS), located approximately 4kb upstream from the miR-200b hairpin [4]. The promoter is sufficient for expression of miR-200b~200a~429 in epithelial cells. MiR-200b~200a~429 gene silencing in mesenchymal cells occurs through the binding of transcriptional regulators, ZEB1 and ZEB2, to specific E-box elements located proximal to the TSS [4–9]. Recently, we and others have shown that the promoter is subject to Polycomb Group (PcG)-mediated gene repression via recruitment of the EZH2 and SUZ12 subunits [33–36]. Unlike most other miRNA genes, miR-200b~200a~429 resides within an intergenic region that has a higher than average GC content (>60%). The genomic architecture of the locus, which comprises repetitive DNA elements, CpG islands, and DNaseI hypersensitivity sites, is characteristic of PcG target genes but also suggest that additional regulatory sequences might exist. This is highly likely given the spatiotemporal characteristics of miR-200b~200a~429 expression during development and its well established functions in epithelial cell biology.

To further understand the transcriptional control of miR-200b~200a~429 in epithelial cells, we have identified novel sequences within the locus that regulate its expression. An enhancer region was identified ~5.1kb upstream of the miR-200b~200a~429TSS, which increased miR-200b~200a~429 expression in epithelial cells. The enhancer was transcribed in breast epithelial and mesenchymal cell lines. The production of the enhancer-transcribed RNA transcript, referred to as *miR-200b eRNA*, was detected in breast epithelial and mesenchymal cell lines, as well as numerous different cell types including fibroblasts and hematopoietic cells, and did not correlate with the miR-200b~200a~429 expression pattern. Our results indicate that the miR-200b~200a~429 enhancer is important for the level of miR-200b~200a~429 expression in breast epithelial cells.

## Results

### An active chromatin domain upstream of miR-200b~200a~429 functions as an enhancer in breast cancer cells

**Figure 1:**
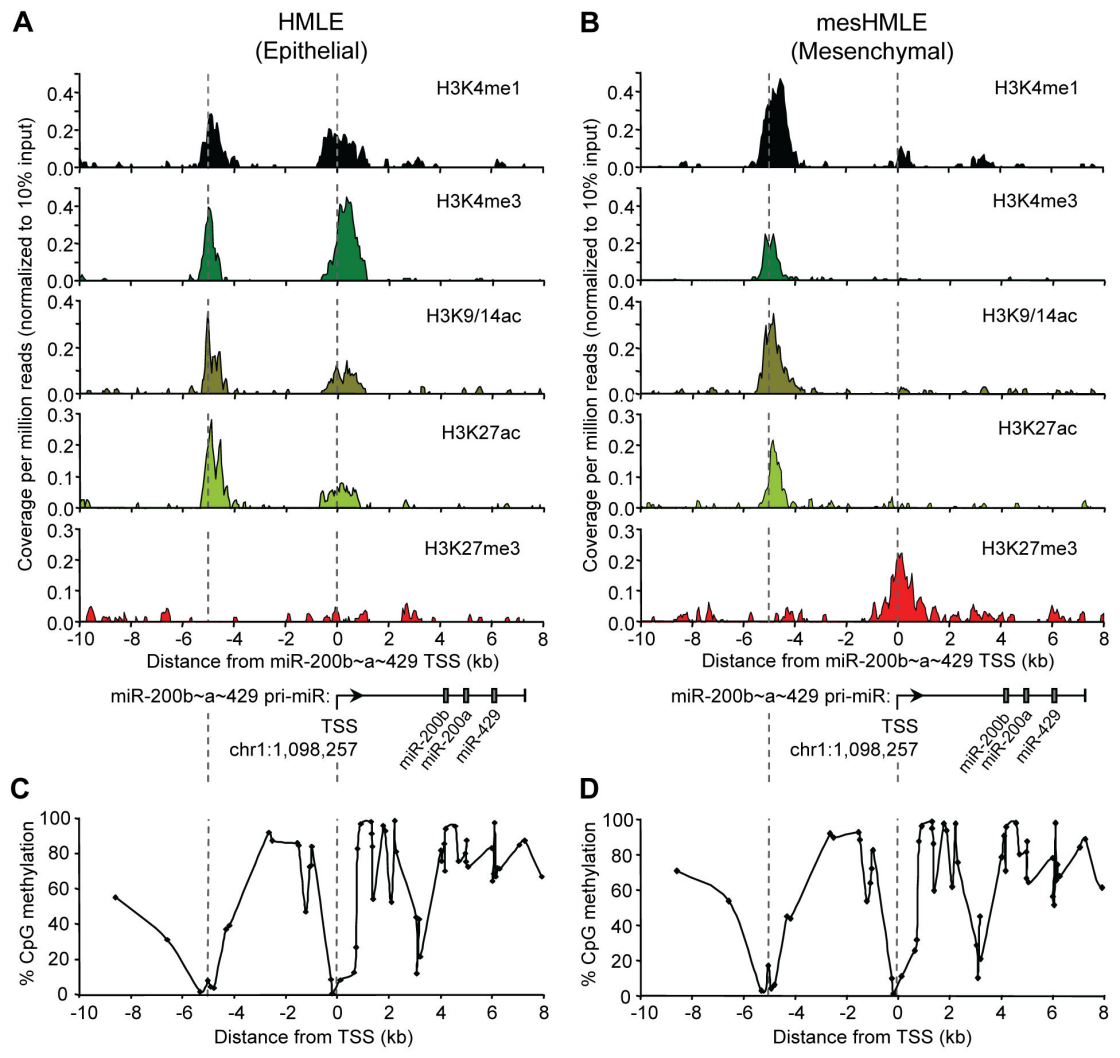
An active chromatin domain is located upstream of the miR-200b~200a~429 locus. Normalized ChIP-seq signal profiles were generated for H3K4me1, H3K4me3, H3K9/14ac, H3K27ac and H3K27me3 at the miR-200b~200a~429 locus (chr1:1,090,000-1,105,000) in (A) epithelial HMLE cells and (B) mesenchymal HMLE that have undergone EMT. The x-axis shows the distance in kilobases (kb) relative to the TSS (designated 0), the chromosomal coordinate marking the TSS is indicated and a schematic diagram of the primary miR-200b~200a~429 transcript is positioned boxes indicate the mature miRNA hairpin transcripts. The y-axis shows the sequencing coverage per million reads for each histone modification normalized to the Input control sample. (C) CpG methylation analysis of the miR-200b~200a~429 locus in epithelial HMLE cells (left panel) and in mesenchymal HMLE cells following 46 days of TGF-β1 (right panel) treatment using Illumina HM450K methylation array [36]. An arrow marks the TSS. The x-axis indicates the distance in kb from the TSS designated 0. The y-axis shows the % CpG methylation occurring at each genomic region.

Given that the miR-200b~200a~429 locus comprises a CpG island located approximately 5 kilobases (kb) upstream of the TSS of miR-200b~200a~429, we hypothesized that it might function as an enhancer element. We therefore examined whether the region surrounding this particular CpG island was associated with the enhancer chromatin signature [16,18,21,23]. HMLE human mammary epithelial cells maintained in HuMEC media were induced to undergo EMT by culturing them in serum-containing media and Transforming Growth Factor-β1 (TGF-β1) for approximately 14 days [2,36,37]. During this 2 week period, HMLE cells lost their epithelial traits and acquired mesenchymal characteristics showing increased cell motility and migration, and an EMT gene signature. These cells were referred to as mesenchymal HMLE (mesHMLE) cells. Chromatin immunoprecipitation assays coupled to next generation sequencing (ChIP-seq) was carried out on the HMLE and the mesHMLE cells and analysis of the data revealed the expected correlation of activating and silencing histone modifications with gene expression at the miR-200b~200a~429TSS on chromosome 1 [36]. At approximately 5 kb upstream of the TSS, we detected the presence of an active enhancer chromatin signature comprising H3K4me1, H3K4me3, H3K9/14ac and H3K27ac in both cell types. Intriguingly, the H3K27me3 mark that covered the TSS in mesHMLE cells did not spread into the active enhancer-like chromatin domain, suggesting the region remained in an accessible chromatin configuration following miR-200b~200a~429 gene silencing (Figure 1A-B). This notion was further supported by the observation that CpG methylation was absent at the upstream region (Figure 1C-D). The active enhancer chromatin domain was confirmed in independent ChIP-qPCR assays (Figure S1A-B). Similar results were observed in the MDA-MB-468 (epithelial) and MDA-MB-231 (mesenchymal) cell lines indicating that the enhancer chromatin signature was not specific to the HMLE cell lines (Figure S1).

**Figure 2:**
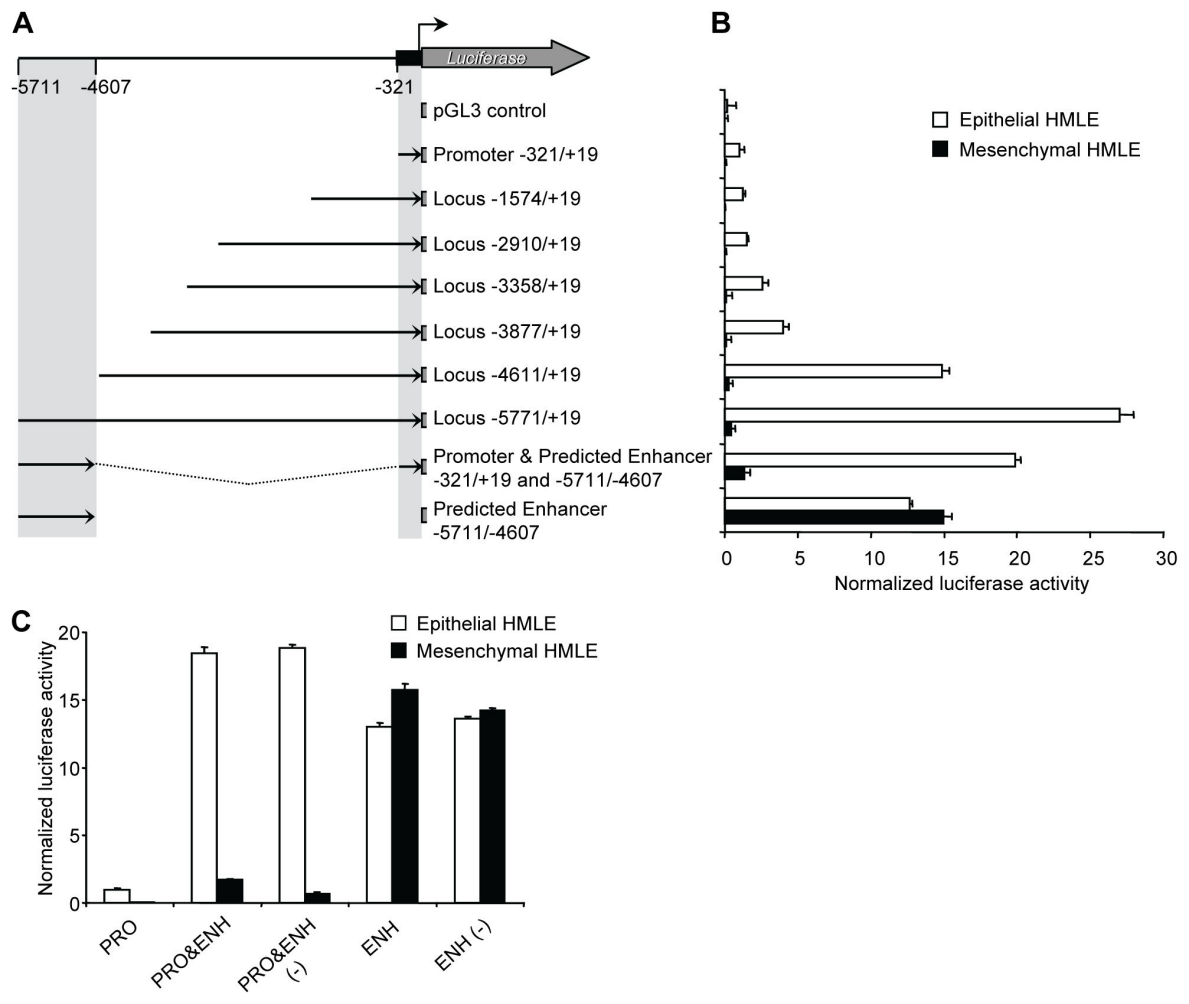
Identification of an upstream enhancer region that increases the transcription of the miR-200b~200a~429 promoter in epithelial breast cancer cells. (A) A series of 5’ deletions of the human miR-200b~200a~429 locus comprising the promoter and potential enhancer were cloned into a firefly luciferase reporter plasmid. (B) The reporter plasmids were transiently transfected along with the Renilla pTK vector into epithelial HMLE cells (white bars) or mesenchymal HMLE cells (black bars). Luciferase activity was assayed approximately 48 hours later using the Dual-Luciferase Reporter Assay System (Promega). Data are expressed as normalized luciferase activity and represent means ± SD of at least four independent experiments. (C) The enhancer region (-5771/-4607 ENH) was cloned in both directions immediately upstream of the minimal miR-200b~200a~429 promoter (-321/+19 PRO) or the Luciferase coding region creating PRO&ENH and ENH as well as PRO&ENH (-) and ENH (-) with ENH oriented in the sense and antisense orientation, respectively. Luciferase activity was assay as described in (B).

To determine whether this region was acting as a miR-200b~200a~429 enhancer element, we performed transient dual reporter assays using a panel of truncated miR-200b~200a~429 genomic segments driving firefly luciferase expression. As expected, the minimal promoter region (-321 to +19 relative to the TSS) produced luciferase activity in HMLE cells but had little or no activity in the mesHMLE cells (Figure 2A-B) [38]. In order to identify the location of the predicted enhancer element, a series of reporter constructs that spanned upstream to -5771 relative to the TSS were created (Figure 2A). Insertions to -3877 had little effect on activity over levels observed for the minimal promoter, but the larger genomic segments, -4611/+19 and -5771/+19, resulted in higher levels of transcriptional activity. The larger constructs, -5771/+19 and -4611/+19 respectively, provided ~27 fold and ~15 fold higher activity compared to the construct containing only the minimal promoter region. Therefore, the enhancer could be mapped between -5771 and -4611 relative to the TSS, with some enhancer activity also observed between -4611 and -3877. We then cloned the predicted enhancer (-5771/-4607 ENH) in both orientations (sense and anti-sense) upstream of the minimal miR-200b~200a~429 promoter region to create PRO&ENH and PRO&ENH (-) constructs. Both constructs generated a higher level of reporter activity (~ 20 fold) compared to the minimal promoter region (-321/+19 PRO) (Figure 2B-C), and this activity was observed only in the epithelial HMLE cells. Reporter genes in which the predicted enhancer (-5771/-4607 ENH) placed in either direction upstream from the luciferase gene (ENH and ENH(-)) were also tested for activity. Consistent with other previously described enhancers, the constructs showed similar activity suggesting that the enhancer also acts as a bi-directional promoter (Figure 2C) [28,30,39,40]. Interestingly, the enhancer activity was detected in both cell types suggesting that the minimal promoter was responsible for epithelial-specific transcription while the enhancer further increased transcriptional activity of the promoter in these cells. Taken together, these experiments confirmed that a constitutive enhancer exists at ~5.1kb upstream of the miR-200b~200a~429 minimal promoter and contributes to the activity of the miR-200b~200a~429 promoter in an orientation-independent manner.

### *miR-200b* eRNA is transcribed from the enhancer region of the miR-200b~200a~429 locus

**Figure 3:**
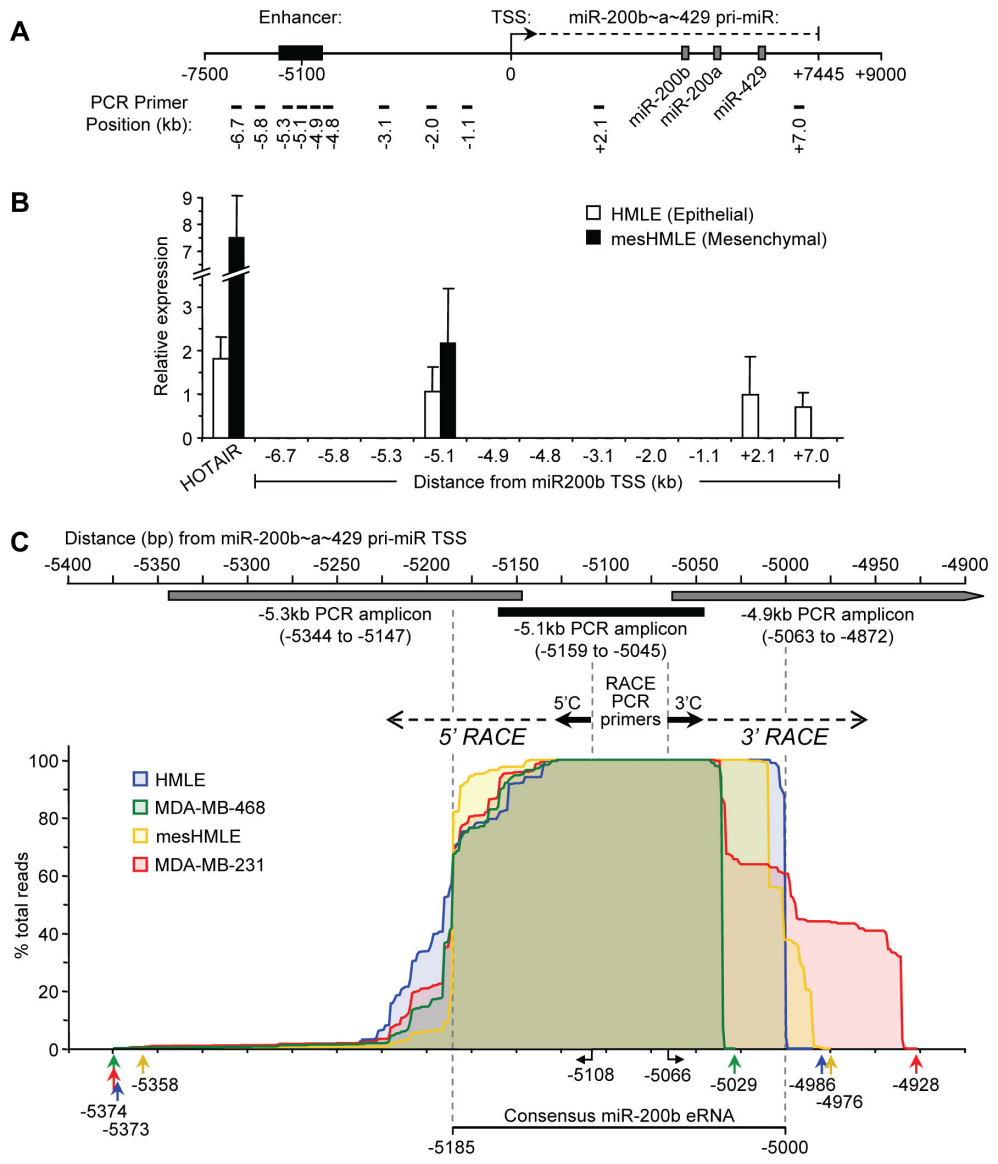
*miR-200b eRNA* is transcribed from the upstream intergenic enhancer region of miR-200b~200a~429. (A) Schematic representation of the miR-200b~200a~429 locus. Black box indicates the position of the potential enhancer. A black arrow marks the TSS direction of the primary miR-200b~200a~429 transcript. Grey boxes indicated the mature miR-200b, miR-200a and miR-429 genes. Black bars indicate the positions (in kilobases, kb) of the PCR primers used for qRT-PCR. (B) Expression levels of HOTAIR, *miR-200b* *eRNA* and the primary miR-200b~200a~429 transcript as determined by qRT-PCR in epithelial and mesenchymal HMLE cells using random hexamer primed cDNA synthesized from total RNA. The x-axis shows the distance from the miR-200b~200a~429 TSS in kb. Data represents mean ± SD of three independent experiments. (C) Schematic representation of the enhancer region located relative to the miR-200b~200a~429 TSS. Boxes indicate the locations of PCR amplicons used to detect the *miR-200b* *eRNA* in Figure 3B. RACE PCR primers and their start locations relative to the miR-200b~200a~429 are indicated. 5’ and 3’ RACE-seq analysis of the *miR-200b* *eRNA* with cDNA prepared from total RNA of HMLE, mesHMLE, MDA-MB-468 and MDA-MB-231 cells as described in the Materials and Methods. 5’ and 3’ ends of the *miR-200b* *eRNA* transcript are mapped as % total reads for each cell line with extreme 5’ and 3’ ends indicated by colored arrows below. A consensus *miR-200b* *eRNA* transcript is indicated.

The observation that the miR-200b~200a~429 enhancer acted as a promoter in the HMLE and mesHMLE cells, prompted us to investigate whether this region was also capable of producing RNA. Indeed, enhancers have shown evidence of non-coding RNA production (>200 nucleotides), termed enhancer RNA (eRNA), in a diverse range of mammalian cell types including neural progenitors, human cell lines, and embryonic stem cells [21,25,41,42,28]. To investigate this possibility, we performed qRT-PCR analysis to search for transcripts derived from the region surrounding the enhancer (Figure 3A). A RT-PCR product was obtained from HMLE and mesHMLE cell cDNA primed with random hexamer but not oligo dT, using primers designed at position -5.1kb upstream of the TSS of miR-200b~200a~429 (Figure 3B and data not shown). Furthermore, this analysis revealed that a significant level of transcription occurred at the -5.1kb position whereas the surrounding intergenic regions contained no detectable transcripts by PCR except for the miR-200b~a~429 pri-miR. Furthermore, the expression level of the transcript while low, was similar to the well-characterized long noncoding RNA, HOTAIR as well as the pri-miR-200b~200a~429 transcript (Figure 3B). Quantitative RT-PCR analysis of cDNA synthesized with strand specific gene specific primers for the enhancer region revealed the production of both sense and antisense RNA transcripts in the HMLE and mesHMLE total RNA fractions (Figure S2). We detected increased amounts of the sense transcript (~5 and ~8 fold higher in HMLE and mesHMLE cells, respectively) compared to the antisense transcript. This analysis revealed bidirectional RNA production at the enhancer and in both cell types, and an overrepresentation of the sense transcript.

To locate the start and end of the major sense transcript we performed 5’-RACE PCR and 3’-RACE PCR on DNaseI-treated total RNA isolated from HMLE and mesHMLE cells, and also from MDA-MB-468 and MDA-MB-231 breast cancer cells. Because the RT-PCR we had performed indicated the transcript was not polyadenylated, we poly(A) tailed the RNA in vitro, prior to the 3’ RACE analysis. To efficiently identify the transcript start and end sites in the various cell lines, we prepared a bar code library using the combined RACE products from each cell line, and sequenced them as a pool on an Ion Torrent sequencer (RACE-seq) (Figure S3). We obtained a total of 293,076 reads containing 29.53Mbp of sequence. Approximately 63% of the reads could be correctly assigned to a barcode/primer following the removal of incomplete reads, and of these, 96.8-98.7% mapped to the expected region on chromosome 1 (Figure 3C). This analysis showed the most frequent TSS in all 4 cell lines corresponded to the C located 5185 bp upstream of the miR-200b~200a~429 pri-miR start site, but with additional heterogeneous start sites arising nearby. The transcript 3’ end was more homogeneous but varied somewhat between cell lines, being located at -5000 relative to the miR-200b~200a~429 pri-miR start site in HMLE cells, but at other nearby locations in the other cell types (Figure 3C). The novel RACE-seq technique employed here, unlike more traditional RACE techniques involving sequencing of individual clones, revealed a comprehensive collection of transcripts of variable transcription start and end sites. The multiple start sites for the transcript were consistent with previous observations showing that CpG island promoters were associated with more than one initiation motif [43,44]. BLAST searches revealed no sequence similarity to any other regions of the human genome. No polyadenylation signals were present in the vicinity of the 3’ ends, consistent with the observation that the transcript could be reverse transcribed using random primers but not oligo(dT) primer (Figure 3C and data not shown). The transcripts were GC rich, revealing an average GC content of 63% (Figure S4). This analysis thus revealed the presence of a RNA transcript of variable size but with a consensus of ~180 nucleotides in human epithelial and mesenchymal cell lines. We refer to this transcript as *miR-200b eRNA* to acknowledge its production from the -5.1kb miR-200b~200a~429 enhancer element.

### Lack of correlation between *miR-200b* eRNA and miR-200b~200a~429 expression patterns

**Figure 4:**
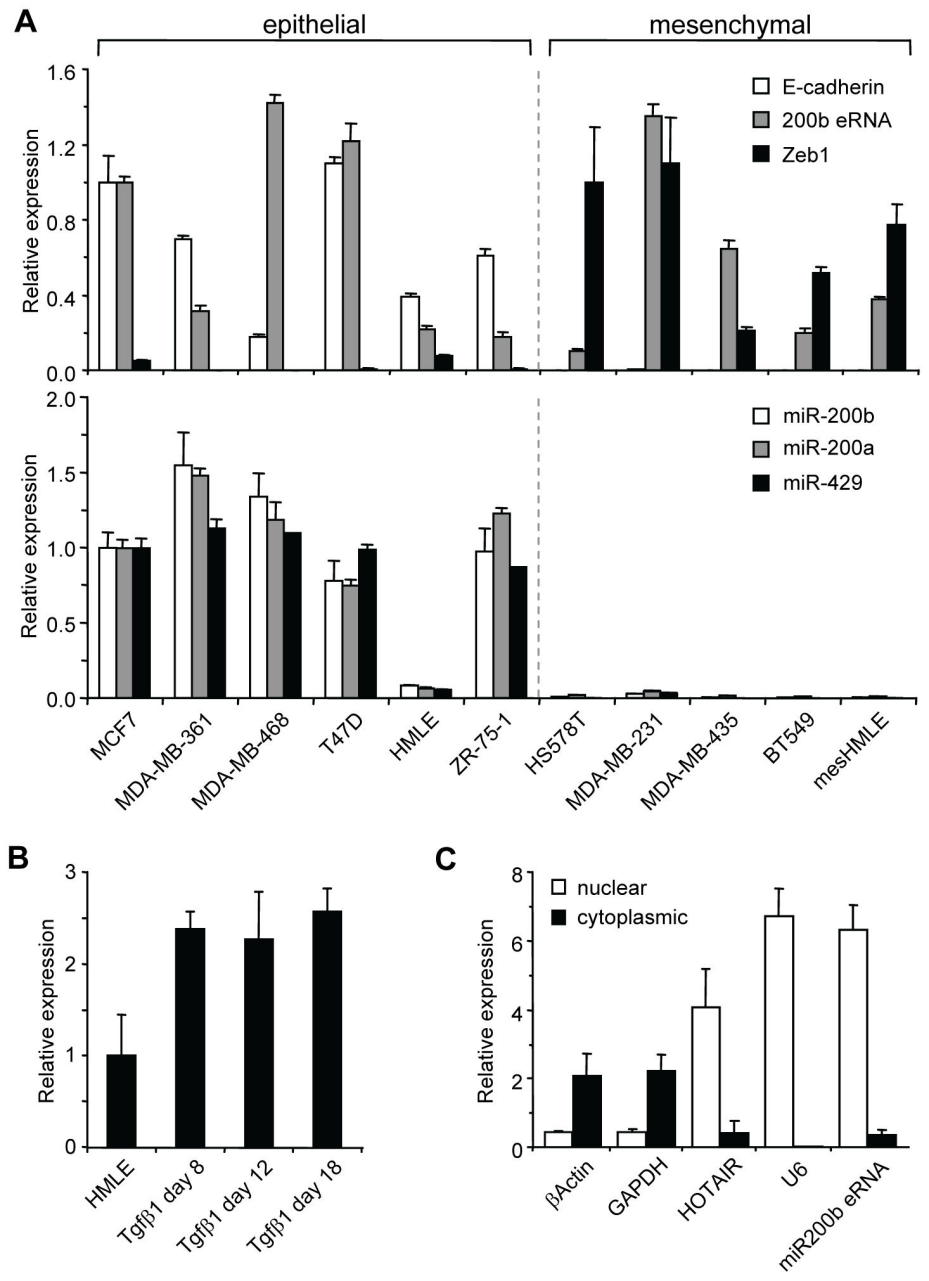
Gene expression analysis of *miR-200b eRNA*, miR-200b~200a~429 and EMT-affiliated genes. (A) Real-time PCR of *miR-200b* *eRNA*, EMT markers and miR-200 genes in a panel of breast epithelial and mesenchymal cell lines. mRNA (top panel) is normalized to GAPDH while miRNA (bottom panel) is normalized to U6 snRNA. Comparative quantitation was used to determine expression profiles of the genes in the cell line panel. Expression of E-cadherin and *miR-200b* *eRNA* in HMLE cells is set to a value of 1, whereas Zeb-1 is expressed relative to mesHMLE cells having a value of 1. Error bars represent mean ± SD of three independent experiments. (B) Relative expression levels of *miR-200b* *eRNA* during EMT time course of HMLE cells treated with TGF-β1 for up to 18 days. The realtime PCR data is normalized to GAPDH. Expression of *miR-200b* *eRNA* is set to a value of 1 in HMLE cells (C) Relative concentration of *miR-200b* *eRNA*, U6, HOTAIR, GAPDH and βActin RNA transcripts in the nucleus and cytoplasm of HMLE cells. Absolute quantitation was used to determine the expression level of each gene in the cytoplasmic and nuclear fractions. Error bars represent mean ± SD of three independent experiments.

Although the relationship between enhancer function and transcription across an enhancer is currently unclear, there is some evidence that eRNAs are functional and have roles in regulating transcription of genes *in cis* [41]. It has been shown that eRNAs can affect transcription both positively and negatively [31]. We therefore compared the expression pattern of *miR-200b eRNA* with miR-200b~200a~429 gene cluster and other epithelial (E-Cadherin) and mesenchymal (ZEB1) genes in a panel of breast cancer cell lines. We used GAPDH as a normalization control gene because the levels of this housekeeping gene did not change in EMT (Figure S5). As shown in Figure 4A, *miR-200b eRNA* was expressed at varying levels in the epithelial and mesenchymal cell types. By contrast, the miR-200b~200a~429 and E-Cadherin genes were restricted to epithelial cell lines, while the mesenchymal cell types exclusively expressed ZEB1. Consistent with other described eRNAs, the expression level of *miR-200b eRNA* was low (Ct values 26-28) [25,41]. In contrast, the expression levels of the ZEB1 and E-cadherin mRNA transcripts were substantially higher (13 and 112 fold respectively) with Ct values ranging from 20-24 using equally efficient qRT-PCR primers. Thus, our analysis revealed that *miR-200b eRNA* did not correlate with the miR-200b~200a~429 gene cluster expression pattern.

We next determined whether the expression level of *miR-200b eRNA* changed during EMT as modeled by the HMLE *in vitro* system [2,37]. At various time points during the 2 week transition period, cells were harvested and analyzed for changes in expression of the *miR-200b eRNA* transcript (Figure 4B). We found that the expression level increased approximately 2 fold following exposure to TGF-β1 for 8 days and this level was maintained in the longer term mesenchymal cultures (>18 days of exposure to TGF-β1). To further investigate the role of *miR-200b eRNA* in transcriptional regulation, we performed sub-cellular fractionation of the HMLE and mesHMLE cells. The analysis revealed that the *miR-200b eRNA* was predominately found in the nuclear fraction rather than the cytosol (Figure 4C). This result was consistent with recent publications showing eRNAs are predominately located in the nucleus [41]. Taken together, these results indicated that *miR-200b eRNA* is a nuclear non-coding RNA that is weakly induced during an early phase of transition to the mesenchymal cell state.

### Analysis of the effect of *miR-200b* eRNA on the transcriptional activity of the miR200b~200a~429 promoter region

To investigate the role of *miR-200b eRNA* in gene regulation, we custom designed siRNAs against the predominating *miR-200b eRNA* transcript targeted by four different sequences. Initial experiments were performed to test whether the cellular level of *miR-200b eRNA* was decreased by the siRNAs targeting the transcript but effective knockdown was not achieved (Figure S6). Similar data was obtained for other cell lines including MDA-MB-468, mesHMLE and MDA-MB-231 cells (data not shown). Additional siRNAs could not be designed and tested from other manufacturers due to the high GC content and relatively short sequence length (<200 nucleotides). In the qRT-PCR surveys of different human cell types and tissues, we found that the *miR-200b eRNA* was expressed in all cell types analyzed including human fibroblasts, T cells and bone marrow-derived cells (Figure S7). Thus, we decided to over-express *miR-200b eRNA* and assess the effect of increased levels of *miR-200b eRNA* on miR-200b~200a~429 promoter activity in epithelial and mesenchymal HMLE cells, as well as other breast cancer cell lines.

**Figure 5:**
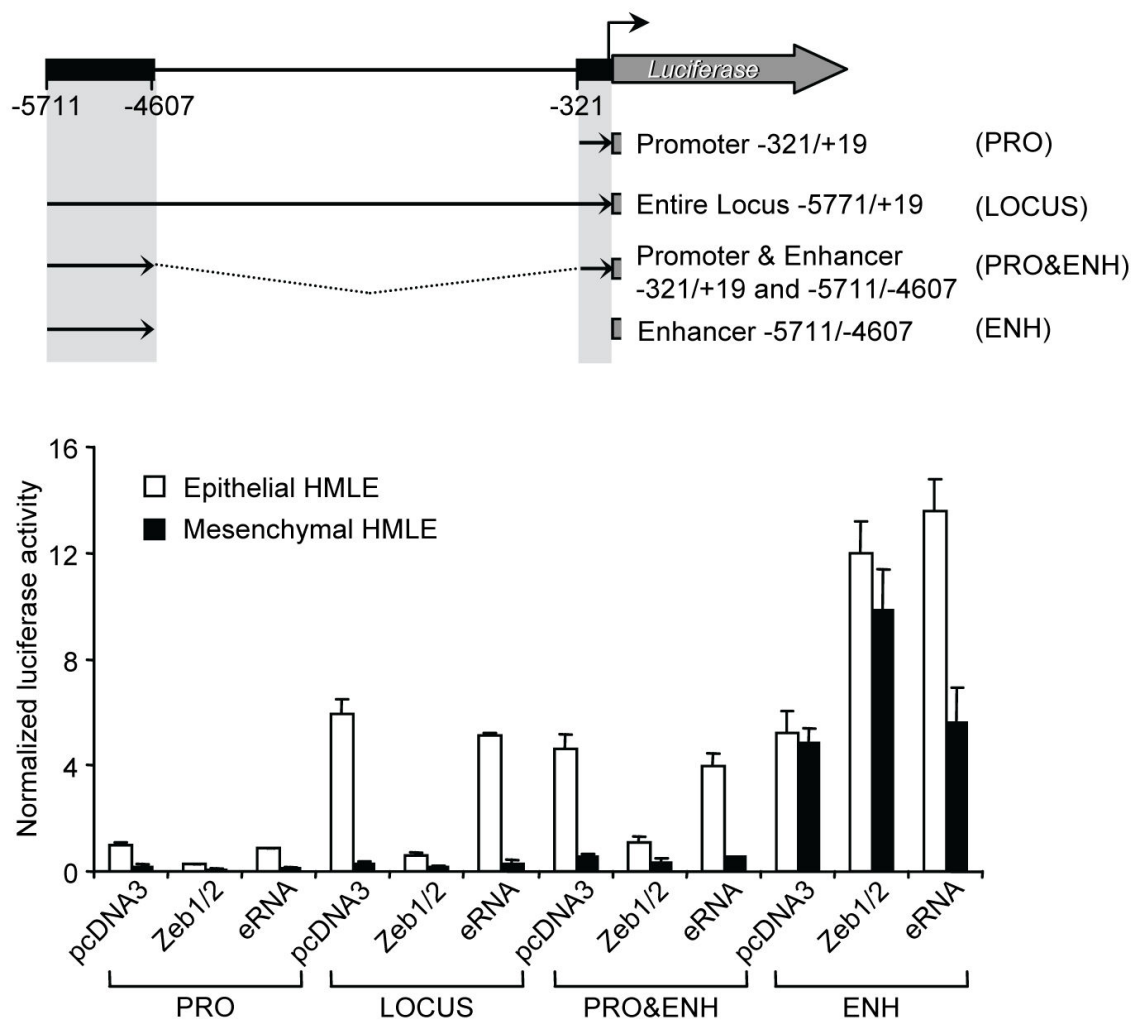
Over-expression of *miR-200b eRNA* has no effect on miR-200b~200a~429 promoter activity in epithelial and mesenchymal HMLE cells. Schematic representation of the miR-200b~200a~429 luciferase reporter constructs, PRO (-321/+19), LOCUS (-5771/+19), PRO&ENH (-321/+19 to -5711/4607) and ENH (-5711/-4607). The miR-200b~200a~429 reporters, pcDNA3.1, pcDNA3.1 Zeb1, pcDNA3.1 Zeb2 or the pcDNA3.1 *miR-200b* *eRNA* plasmids, and the Renilla vector were co-transfected into epithelial HMLE (white bars) or mesenchymal HMLE (black bars) cells. Transiently transfected cells were incubated for approximately 24-48 hours. Data are normalized by Renilla luciferase activity and represent means ± SD of at least three independent experiments.

To investigate whether *miR-200b eRNA* was capable of regulating transcription of miR-200b~200a~429 *in trans*, we conducted luciferase reporter gene assays. For these experiments, we employed the miR-200b luciferase reporter plasmids (Figure 2A), pcDNA over-expression vectors comprising the *miR-200b eRNA* transcript (Materials and Methods) and ZEB1 and ZEB2 expression vectors as a control [4]. Over-expression of *miR-200b eRNA* in HMLE cells resulted in ~100 fold increase in expression compared to endogenous eRNA transcript levels in these cells (Figure S8). In HMLE cells that express miR-200b~200a~429, we found that ZEB1/2 over-expression inhibited miR-200b~200a~429 minimal promoter (PRO) activity, whereas over-expression of *miR-200b eRNA* had little or no effect on PRO activity (Figure 5). Similar trends were observed in the context of the entire locus (LOCUS) and the enhancer fused to the minimal promoter (PRO&ENH). Co-transfection with either the ZEB1/2 or eRNA constructs in mesHMLE cells with the promoter constructs had no effect on reporter activity. By contrast, we found that ZEB1/2 over-expression stimulated the activity of the enhancer-only (ENH) reporter construct in both HMLE and mesHMLE cells, while *miR-200b eRNA* stimulated the enhancer-only reporter in HMLE but not mesHMLE cells (Figure 5). The reporter gene assays were also conducted in the respective epithelial and mesenchymal cell lines, MDA-MB-468 and MDA-MB-231, which provided similar results (Figure S9). However, in MDA-MB-468 cells, over-expression of ZEB1/2 and *miR-200b eRNA* caused no change in activity of the ENH reporter construct (Figure S9). Furthermore, over-expression of *miR-200b eRNA* in HMLE and mesHMLE cells did not induce EMT/MET nor did it lead to changes in miR-200b~200a~429 and EMT/MET-affiliated gene expression patterns (Figure S10; data not shown).

Overall, these results provide evidence that enforced expression of the *miR-200b eRNA* transcript does not influence miR-200b~200a~429 and EMT-affiliated gene expression patterns nor does it affect the maintenance of the epithelial or mesenchymal cell state. However, we cannot fully rule out the possibility that the transcript regulates these processes because depletion of *miR-200b eRNA* using targeted siRNAs was technically unachievable. Furthermore, exogenous *miR-200b eRNA* transcript was polyadenylated, due the use of pcDNA3.1, which may have influenced the physiological structure and function of the eRNA *in vivo*. Despite this, we conclude based on the experimental data shown here that the miR-200b~200a~429 enhancer element is important for optimal miR-200b~200a~429 promoter activity, and produces a noncoding eRNA transcript, whose function remains to be determined.

## Discussion

Epithelial cells undergo major changes in morphology and migratory capacity as they transition into mesenchymal cells during TGF-β1-mediated EMT. Such changes in cellular function are proposed to result from chromatin reorganization of the genome, allowing for the establishment and maintenance of cell type specific gene expression signatures. A major player in this process is the miR-200 gene family which is required for maintenance of the epithelial cell state but becomes silenced in mesenchymal cells, leading to increased cell motility, proliferation and migration. Here, we have investigated the existence of additional regulatory elements responsible for controlling miR-200b~200a~429 expression in epithelial cells. We identified an H3K4me1-enriched enhancer sequence located roughly 5.1kb upstream of the miR-200b~200a~429TSS, and classified it as an active regulatory element based on its co-association with H3K4me3, H3K27ac and RNAPII. The miR-200b~200a~429 enhancer was functional in epithelial and mesenchymal cell types, and produced a short non-coding RNA, *miR-200b eRNA*, linking eRNA production to enhancer function. These findings indicate that the miR-200b~200a~429 enhancer can increase the expression from the minimal miR-200b~200a~429 promoter. However the miR-200b~200a~429 promoter is responsible for confining expression to epithelial cells.

Recent genome-wide ChIP-seq studies have revealed that gene promoters associate with H3K4me3, whereas H3K4me1 is often found at enhancer elements but shows very little association with H3K4me3 [16,18]. Although the classification of enhancer elements in terms of chromatin modifications is in the early stages, some general features have already emerged. It has been proposed that H3K4me1-enriched enhancers can be classified as either “active” or “poised” by the co-association of additional histone modifications, namely, H3K27ac and H3K27me3, respectively [20,23,24,31]. Active enhancers are typically associated with RNAPII and H3K27ac whereas poised enhancers often contain the repressive H3K27me3 mark but lack RNAPII and H3K27ac. Our results suggest that the miR-200b~200a~429 enhancer is maintained in a transcriptional active or competent state given that the region was occupied by an active enhancer signature in both epithelial and mesenchymal cells. However, it remains to be determined whether these marks are functionally important for miR-200b~200a~429 enhancer activity or are of consequence to the active enhancer state. It is currently unclear how these histone marks are deposited and maintained at enhancers during genomic processes such as nucleosome remodeling, histone variant deposition and rapid histone turnover [45–47]. Nevertheless, it will be interesting to identify transcriptional complexes that interact at the miR-200b~200a~429 enhancer in future work.

More recently, enhancer elements have been shown to produce non-coding RNA transcripts, termed enhancer RNAs (eRNAs) [27,28,48], non-coding RNA-activator (ncRNA-a) [41] and enhancer non-coding RNA (e-ncRNA) [21]. Their defining features include lack of polyadenylation, an average size of 900 nucleotides, and the absence of transcript splicing [29]. At a functional level, enhancer-transcribed RNAs have activating or silencing roles in mRNA gene transcription. Some clear examples include *ncRNA-a7* transcribed from the Snai1 enhancer, *Mistral* noncoding RNA which is produced at the HoxA locus [41,49], HOTTIP encoded at the HoxA enhancer site [48], and eRNAs produced from p53-bound enhancer elements [28]. The *miR-200b eRNA* described here can be classed as a typical eRNA due to its lack of polyadenylation, short length (~180 nucleotides) and lack of transcript splicing. However, the functional role of *miR-200b eRNA* in controlling miR-200b~200a~429 gene expression remains elusive. Knockdown with siRNA was unsuccessful despite numerous attempts at custom design and optimization of transient transfection conditions. It is possible that the presence of antisense *miR-200b eRNA* could have prevented efficient siRNA knockdown of the sense transcript. However, the level of the antisense transcript was lower (~5-8 fold reduced) than the sense *miR-200b eRNA*, and we performed extensive individual and pooled siRNA titrations in order to optimize the targeting of siRNAs to the sense *miR-200b eRNA* transcript. Thus, we concluded that the relatively small target sequence (~180 nucleotides), GC rich sequence content, presence of the antisense transcript and mainly nuclear localization impaired effective knockdown of *miR-200b eRNA* using siRNAs. Improvements in siRNA delivery to the nucleus, custom design software and/or specific modified strand specific DNA oligonucleotides may increase the likelihood of finding an effective knockdown approach for short GC-rich target sequences such as the one described here.

The mechanism of enhancer regulation of gene expression and regulation of enhancer elements themselves is fundamental to understanding gene transcriptional control. Given our finding that the upstream miR-200b~200a~429 enhancer element increases miR-200b~200a~429 expression in epithelial cells, a next key question will be how this element works in concert with the promoter, presumably through chromosomal looping, which could facilitate the recruitment of transcriptional complexes. Another important question will be why the miR-200b~200a~429 enhancer element also behaves like a promoter, producing its own RNA. In this study, the biological function of the *miR-200b eRNA* remained unresolved. However, it is tempting to speculate that the constitutive production of the *miR-200b eRNA* maintains an transcriptionally competent locus during EMT, thus allowing for rapid reactivation of the miR-200b~200a~429 promoter when mesenchymal cells revert back to an epithelial cell state during MET. Further elucidation of the miR-200b~200a~429 enhancer element and *miR-200b eRNA* function should bring about new insights into the role of enhancers and regulation of gene transcription in EMT and breast cancer metastasis.

## Materials and Methods

### Cell line maintenance

HMLE cells were cultured in HuMEC ready media (Gibco) while other human breast cancer cell lines were cultured as previously described [4]. To induce EMT and generate mesHMLE cells, HMLE cells were cultured for at least 14 days in DMEM: F12 media (1:1) supplemented with 10 µg/ml insulin, 20 ng/ml EGF, 0.5 µg/ml hydrocortisone, and 5% fetal calf serum (FCS) and treated with 2.5 ng/ml of TGF-β1 (R&D) [2,36,37].

### Generation of miR-200b~200a~429 promoter and the pcDNA3 miR-200b eRNA constructs

A -5771/+19 genomic region containing the miR-200b~200a~429 promoter [4] was amplified by PCR using *Pfu* DNA polymerase (Promega) and directionally cloned into the pGL3-basic (Promega) reporter using *NheI* and *BglII* sites generating the -5771/+19 construct. Subsequent promoter truncations were amplified from this template and cloned in a similar manner which resulted in the -4611/+19, -3877/+19, -3358/+19, and -2910/+19 constructs. The -321/+19 (PRO) and -1574/+19 constructs were utilized as previously described [38]. The PRO&ENH construct was made by excising intervening sequence (-322 to -4608) from construct -5771/+19 using BamHI sites that resulted in placing the enhancer region (-5711/-4607) in sense and antisense orientation directly upstream to the minimal promoter region (PRO -321/+19). The enhancer only (ENH) -5711/-4607 construct was made by PCR and cloning into pGL3-basic using a single KpnI site in order to create clones with sense and antisense orientation of the enhancer region. The pcDNA3.1 miR-200b eRNA expression construct was made by PCR amplification of the 185 bp eRNA sequence spanning -5209 to -5023 relative to the putative TSS, and directional cloning into pcDNA3.1 using HindIII and BamHI sites. Constructs were confirmed using pyrosequencing of plasmid DNA.

### Transfection and reporter assay

For reporter assays, cell lines were plated in 24 well plates and co-transfected with 5 ng pRL-TK Renilla plasmid (Promega) and 200 ng miR-200b~200a~429 promoter firefly luciferase reporter plasmids using Lipofectamine LTX (Invitrogen). In Figure 5, 100 ng of the pcDNA3.1 empty vector, ZEB1 and ZEB2 expression plasmids (pcDNA3.1-HisC-ZEB1 and pcDNA3.1-HisC-ZEB2; 50 ng each) or 100 ng of the *miR-200b eRNA* expression plasmid (pcDNA3.1-miR-200b eRNA) were also co-transfected. After 48 hr of incubation, cell lines were either lysed in Trizol (Invitrogen) and RNA extracted or lysed in passive lysis buffer (Promega) and luciferase activity measured with the Dual-Luciferase Reporter Assay System (Promega) using a Glomax-multi detection system (Promega). All reporter assays are shown as normalized luciferase activity (averaged ratios of Firefly luciferase:Renilla ± SEM) and are representative of multiple experiments.

### siRNA transfection

For siRNA reporter experiments, individual or mixtures of four siRNAs (Dharmacon) of human *miR-200b eRNA* were transfected into cells using HiPerFect (Qiagen) at 10, 50 and 100nM each, then repeat-transfected 48 hr later with the same siRNAs. RNA was harvested after a further 24-48 hr followed by real-time PCR analysis of random hexamer primed cDNA. Target sequences of the *miR-200b eRNA* siRNA were the following: siRNA#1 GCT GAC TAG AGG AGG CAA A; siRNA#2 CCA GGG TTC TCC AAG CAA A; siRNA#3 CGC CGA GGA GAC TGG GTT TT; and siRNA#4 AAG CAA AGC CTG TCT GTG TT. The ON-TARGETplus non-targeting smart pool negative control (Dharmacon) was used as a negative control.

### Isolation of RNA and real-time PCR

Total RNA was extracted from cell lines and purified using Trizol (Invitrogen). Real-time PCR was performed as previously described [5]. Briefly, for mRNA measurements, cDNA was synthesized using a QuantiTect Reverse Transcription Kit (Qiagen) and real-time PCR was performed using QuantiTect SYBR, Green PCR kit (Qiagen) with primers as listed (Tables S1-S2). MicroRNA PCRs were performed using TaqMan microRNA assays (Life Technologies) (Table S3). Real-time PCR data for mRNA and microRNA were expressed relative to glyceraldehyde 3-phosphate dehydrogenase (GAPDH) or U6 snRNA, respectively. Quantitation was performed using a Rotor-Gene 6000 (Qiagen).

### 5’ RACE

5’ RACE was performed using the 5’ RACE system for Rapid amplification of cDNA Ends, Version 2.0 (Invitrogen) according to the manufacturer’s instructions. Briefly, 3 µg total RNA was treated with DNaseI (Ambion). First strand cDNA synthesis was performed using the 5’ A miR-200b eRNA custom designed gene specific primer. The cDNA was purified using a S.N.A.P. column (Invitrogen) then a poly(C) tail was added to the 3’ end of the cDNA using TdT and dCTP. The dC-tailed cDNA underwent two rounds of PCR amplification using nested eRNA specific primers (5’ B and 5’ C) together with universal amplification primers and Taq Advantage 2 polymerase (Clontech). PCR products were checked on a 2% Agarose gel following each round of PCR amplification. Sequences of the three gene specific primers are listed in Table S4.

### 3’ RACE

3’ RACE was performed using the 3’ RACE system for Rapid amplification of cDNA Ends (Invitrogen) according to the manufacturer’s instructions using the recommended modified method for transcripts with high GC content. Briefly 3 µg of total RNA was treated with DNaseI (Ambion) then polyadenylated using a poly(A) Tailing Kit (Ambion). First strand cDNA synthesis was initiated at the poly(A) tail using an Adapter primer. Three rounds of nested PCR amplification of the *miR-200b eRNA* cDNA was performed with custom designed gene specific primers (3’ A, 3’ B and 3’ C) and Universal Adapter Primers using Taq Advantage 2 polymerase mix (Clontech). PCR products were checked on a 2% Agarose gel following each round of PCR amplification. Sequences of the three gene specific primers are listed in Table S4.

### RACE-seq

A sequencing library was prepared using an Ion Plus Fragment Library Kit (Life Technologies) following the manufacturers recommended protocol with the exception that all PCR product clean-up was column based using a QIAquick PCR purification kit (Qiagen). For each cell line, equal amounts of 5’ & 3’ RACE products were pooled (~50 ng total). The RACE PCR pool was purified using a QIAquick PCR purification kit (Qiagen) then underwent end repair (Ion Plus Fragment Library kit). End repaired RACE PCR products were then purified using QIAquick PCR purification columns before Adapter ligation (Ion Xpress P1 adapter & Ion Xpress Bar code adapters 5-8 from Ion Xpress Bar code Adapters 1–16 Kit) (Life Technologies) using a different bar code adapter for each cell line pool. Ligation was followed by nick repair to complete the linkage between the adapters and DNA inserts using reagents from the Ion plus Fragment library kit. Libraries were purified using the QIAquick PCR purification kit (Qiagen) before being checked for integrity & size on a Bioanalyzer (Agilent Technologies). The libraries were gel purified on a 2% Agarose gel selecting fragments > 150bp to remove contaminating unincorporated adapters, and purified using a QIAquick gel extraction kit (Qiagen). The size fractionated library was then PCR amplified (using primers and reagents from the Ion Plus Fragment Library kit) for 5 cycles to select for P1-X combination of ligated ends suitable for sequencing. The PCR amplified libraries were purified a final time using QIAquick PCR purification kit (Qiagen) before being checked on a Bioanalyzer (Agilent Technologies) and equal amounts of each individual library were combined into a single library pool. The library pool was clonally amplified on Ion Sphere Particles using the Ion PGM 200 Xpress Template kit prior to loading on an Ion 314 chip and sequencing on the Ion Torrent PGM (Life Technologies) using 130 cycles. Sequences obtained from each cell line were identified using their unique bar codes. For each sequencing read, the bar codes were read, trimmed & products put into 4 bins (corresponding to each cell line RACE pool) for subsequent analysis. The Ion Torrent library preparation adapters and the poly(n) sequences and adapters added during the RACE protocol were removed leaving specific sequences corresponding to either 5’ or 3’ RACE products. These sequences were mapped to the human hg19 reference genome, and 5’ or 3’ ends of each transcript were identified. The raw and processed data files can be downloaded from https://bitbucket.org/sacgf/attema_2013_200_enhancer.

### DNA methylation analysis

Genomic DNA was isolated from cells using either DNeasy Blood & Tissue kit (Qiagen), Trizol, or phenol chloroform ethanol precipitation method (Invitrogen). 0.5 to 2 µg genomic DNA was bisulphite modified with the EZ DNA Methylation-Gold Kit according to the manufacturer’s protocol (Zymo Research). Bisulphite-modified genomic DNA was used for hybridization on Infinium HumanMethylation 450 BeadChip, following the Illumina Infinium HD Methylation protocol, and the BeadChip was scanned using an Illumina HiScan SQ scanner (Illumina, San Diego, CA, USA). The methylation score for each CpG was represented as a β-value according to the fluorescent intensity ratio. β-values may take any value between 0 (non-methylated) and 1 (completely methylated).

### ChIP-qPCR

ChIP assays using 1 x 10^6^ cells per reaction were performed as recently described [36]. Antibodies included anti-histone H3 (ab1791; Abcam), and anti-trimethyl histone H3K4 (ab8580; Abcam), anti-monomethyl histone H3K4 (ab8895; Abcam), anti-acetyl histone H3K9/14 (06-599; Millipore), anti-acetyl histone H3K27 (07-449; Millipore), anti-trimethyl histone H3K27 (abcam ab6002) and anti-RNA Polymerase II Phospho S2 and S5 (ab5131; ab5095; Abcam). Briefly, 6 x 10^6^ cells were crosslinked in 1% formaldehyde for 10 min at room temperature with gentle rocking or inversion every 2-3 min. Cells were quenched with 0.25M Glycine, pelleted by centrifugation (300 rcf for 5 min), and washed twice in ice-cold 1x HBSS (Gibco) containing protease inhibitor cocktail (Roche). The cells were lysed in 300 µl of lysis buffer (10 mM Tris pH 7.5/1mM EDTA/1% SDS) containing PIC and incubated on ice for 10 min. After lysis, 900 µl of 1x HBSS containing PIC was added and 200 µl was aliquoted into 6 individual tubes. Each 200 µl aliquot was sonicated by using a bioruptor® sonicator (Diagenode), which was empirically determined to give rise to genomic fragments ~500bp. The soluble chromatin was collected by 4°C ultracentrifugation (13,000 rpm for 5 min) and pooled into a new 15ml falcon tube. The supernatant was diluted 2-fold with 2x RIPA buffer (10 mM Tris-HCl pH 7.5; 1 mM EDTA; 1% Triton X-100; 0.1% SDS; 0.1% sodium deoxycholate; 100 mM NaCl; PIC), 1/10 volume (40 µl) input was removed, and 400 µl of soluble chromatin (equivalent to 1 x 10^6^ cells) was distributed to new Eppendorf tubes. Each respective antibody was added at appropriate amount as tested in titration experiments using control promoters. Immunoprecipitations were performed for 2 h at 4°C with rotation, and antibody: protein:DNA complexes were then collected with 50 µl of protein A and/or G Dynabeads (Invitrogen) for 2 h of rotation. The beads were washed three times using 200 µl of RIPA buffer and once with TE buffer, then incubated with 200 µl of fresh elution buffer with Proteinase K for 2 h in a thermomixer (1300 rpm, 68°C) to reverse the protein:DNA cross-links. After incubation, eluates were collected into new eppendorf tubes. Genomic DNA was recovered by using phenol chloroform extraction and ethanol precipitation. Pellets were washed in 70% ethanol, briefly air-dried, and resuspended in TE (10 mM Tris pH 7.5; 0.1 mM EDTA) buffer. Quantitation of ChIP DNA (relative enrichment) was performed using a Rotor-Gene 6000 (Qiagen) with QuantiTect SYBR green PCR Kit (Qiagen) and ChIP qPCR primer sequences as listed in Table S5. Enrichment of histone modifications at genomic regions were expressed as % input normalized to respective cell type histone H3 levels. % Input was calculated using the formula % (ChIP/Input) = 2^(Ct(ChIP) - Ct(Input))^ x Input Dilution Factor x 100% to account for chromatin sample preparation differences.

### ChIP-seq

For ChIP-seq experiments, the individual or pooled (n=3) ChIP samples were submitted to Geneworks Pty Ltd (Adelaide) for the ChIP-seq full service (library preparation and sequencing). The library assembly was performed using the TruSeq ChIP sample preparation kit (Illumina Inc.). ChIP DNA libraries comprising single-ended 65bp long fragments directly sequenced using a Genome Analyzer IIx (Illumina Inc.) resulting in >20 million reads per sample. Sequencing reads were aligned to the hg19 build of the human genome and duplicate sequences were removed. Genomic DNA coverage across the miR-200b~200a~429 locus (chr1:1,090,000-1,105,000) was calculated as follows: read coverage of experimental ChIP samples were calculated per base pair (bp), normalized by sequencing depth, and subtracted from the corresponding 10% Input control sample. Average coverage was calculated over 50 bp regions. The ChIP-seq data used for this study was extracted from a region of chromosome 1 (chr1:1079434-1109285). The raw and processed data files can be downloaded from https://bitbucket.org/sacgf/attema_2013_200_enhancer.

### Sub-cellular fractionation

Nuclear/cytoplasmic fractionation of epithelial and mesenchymal HMLE cells was performed using hypotonic buffers. Briefly, approximately 1 x 10^7^ cells were harvested by trypsin, washed with phosphate buffered saline (PBS) and incubated for 10 min on ice in 900 µl of Hypotonic Buffer (10 mM HEPES-KOH (pH7.9), 10 nM KCl, 1.5 mM MgCl_2_, 1 mM DTT, protease inhibitor cocktail tablet (Roche), and RNasin (Ambion)). Forty five microlitres of 10% NP-40 was added, and cells were pipetted up and down with a p1000 pipette 60 times, and incubated on ice for 5 min. Cells were microcentrifuged for 5 mins at 4000 rpm at 4°C. Supernatant comprising the cytoplasmic fraction was collected and stored at -80°C until further processing for total RNA or protein. The nuclei pellet was then washed in 500 µl of Hypotonic Buffer three times and finally resuspended in 300 µl nuclear lysis buffer (20 mM HEPES-KOH (pH7.9), 400 mM NaCl, 1.5 mM MgCl_2_, 0.2 mM EDTA, 1 mM DTT, 5% glycerol, protease inhibitor cocktail (Roche), and RNasin (Ambion), and incubated for 30 min on ice. The nuclear pellet was microcentrifuged for 10 min at 13000 rpm at 4°C and the supernatant containing the nuclear fraction was collected and stored at -80°C until further processing for total RNA or protein. The concentration of total RNA derived from the nuclear and cytoplasm were quantified using the Nanodrop ND-1000 spectrometer (Nanodrop Technologies) and normalized prior to cDNA synthesis using Superscript III Reverse Transcriptase (Invitrogen). The amount of cDNA in each fraction was determined by real-time PCR using a Rotor-Gene 6000 (Corbett Life Science) with QuantiTect SYBR green PCR Kit (Qiagen). Real-time PCR primers are listed in Table S1.

## Supporting Information

Figure S1 **An active chromatin domain upstream of the miR-200b~200a~429 locus confirmed by ChIP-qPCR analysis.** Representative ChIP-qPCR analysis of H3K4me1, H3K4me3, H3K9/14ac, H3K27ac and H3K27me3 at the miR-200b~200a~429 locus (chr1:1,090,000 -1,105,000) in (A) epithelial HMLE, (B) mesHMLE, (C) MDA-MB-468 and (D) MDA-MB-231. The x-axis shows the distance up and downstream relative to the TSS and a schematic diagram of the primary miR-200b~200a~429 transcript is positioned to scale. An arrow marks the TSS and shaded boxes indicates the mature miRNA transcripts. The y-axis shows % Input of histone H3 modifications normalized to unmodified histone H3.
(TIF)

Figure S2 **The miR-200b~200a~429 enhancer region produces sense and antisense eRNA transcripts.** Total RNA was isolated from HMLE and mesHMLE cells. Following DNaseI treatment, total RNA was converted to cDNA using random hexamers, 5’ A RACE primer (complementary to the sense RNA transcript) or 3’ A RACE primer (complementary to the antisense RNA transcript) (Table S4). Real-time PCR analysis of cDNA was performed using gene specific primers for the eRNA (Table S1). GAPDH was used for normalization and data was analyzed using the comparative quantitation method shown as relative expression to HMLE random hexamer primed cDNA (set to 1). Error bars represent mean ± SD of two independent experiments.
(TIF)

Figure S3 **Schematic of the 5’ and 3’ RACE-seq methodology.** The RACE-seq method comprises three steps, 5’ and 3’ RACE, Library preparation and Sequencing. DNaseI-treated total RNA isolated from HMLE, mesHMLE, MDA-MB-231 and MDA-MB-468 was subjected to 5’ RACE by incorporating three rounds of nested PCR using gene specific primers (Table S3) (Step 1). 3’ RACE was performed in a similar manner except that the DNaseI-treated total RNA was first polyA tailed (*E. coli* Poly(A) Polymerase I) (Step 1). 5’ and 3’ RACE PCR products obtained from each cell type were pooled into single reaction tube and subjected to library preparation (Step 2). Individual libraries comprising RACE products from each cell line (total of 4) were prepared using sample-specific bar code adapters were then combined and sequenced together (Step 3). Sequences obtained from each cell type were identified using their unique bar codes. For each sequencing read, the bar codes were read, trimmed and sorted into 4 bins (corresponding to each cell line RACE pool). The Ion Torrent library preparation adapters (pink bars) and the poly(n) sequences and adapters added during the RACE protocol (grey bars) were removed, leaving behind specific sequences corresponding to either 5’ or 3’ RACE products (black bars with either a red dot or blue dot representing the respective transcript ends). These sequences were mapped to the human hg19 reference genome and 5’ or 3’ ends were identified.
(TIF)

Figure S4 **Schematic of the *miR-200b eRNA* transcript and its genomic location on human chromosome 1.** The major 5’ and 3’ RACE-seq transcript occurring in epithelial HMLE and mesHMLE cells is shown inset to the location of the transcript produced at enhancer region on human chromosome 1 (hsa chr1:1,092,994-1,093,179). The GC content is indicated.
(TIF)

Figure S5 **GAPDH is suitable for use as a normalization control gene in the HMLE EMT cell line model.** Relative expression levels of the housekeeping genes GAPDH, βActin and β2-microglobulin in the HMLE and mesHMLE cells. Following DNaseI treatment, the RNA was converted to cDNA using random hexamers. Real-time PCR analysis of cDNA was performed using gene specific primers. The data was analyzed using the comparative quantitation method and is shown as relative expression to HMLE (set to 1) for each mRNA tested. Error bars represent mean ± SD of two independent experiments.
(TIF)

Figure S6 **Custom designed siRNAs fail to knock down *miR-200b eRNA* transcript.** Four custom siRNAs were tested in transient transfection assays for their ability to knockdown *miR-200b* *eRNA* in HMLE cells. Individual and pooled siRNAs (1-4) were assayed at 10 nM (top panel), 50 nM (middle panel) and 100 nM (bottom panel). Following DNaseI treatment, the RNA was converted to cDNA using random hexamers. Real-time PCR analysis of cDNA was performed using gene specific primers for *miR-200b* *eRNA* (Table S1). Quantitative RT-PCR data is calculated using the comparative quantitation method and is shown as relative expression to the control siRNA (set to 1) following GAPDH normalization. Error bars represent mean ± SD of three independent experiments.
(TIF)

Figure S7 **Gene expression analysis of *miR-200b eRNA* in other cell types.** Total RNA was isolated from HMLE, mesHMLE, normal bone marrow cells (samples 1-3), W1-38 fibroblast cell line and the Jurkat T cell line. Following DNaseI treatment, the RNA was converted to cDNA using random hexamers. Real-time PCR analysis of cDNA was performed using gene specific primers for *miR-200b* *eRNA* (Table S1). GAPDH was used for normalization. Data was analyzed using the comparative quantitation method and is shown as relative expression to HMLE (set to 1). Error bars represent mean ± SD of three independent experiments.
(TIF)

Figure S8 **HMLE cells transiently transfected with the pcDNA *miR-200b eRNA* plasmid results in ~100 fold increased expression level.** Relative expression using the comparative quantitation method of *miR-200b* *eRNA* in HMLE cells set to 1 (non-transfected), HMLE cells transiently transfected with the pcDNA control vector or the pcDNA *miR-200b* *eRNA* overexpression vector. GAPDH was used for normalization, and error bars represent mean ± SD of three independent experiments.
(TIF)

Figure S9 **Over-expression of *miR-200b eRNA* has no effect on miR-200b~200a~429 promoter activity in MDA-MB-468 and MDA-MB-231 breast cancer cell lines.** Schematic representation of the miR-200b~200a~429 reporter constructs, PRO (-321/+19), LOCUS (-5771/+19), PRO&ENH (-321/+19 to -5711/4607) and ENH (-5711/-4607). The miR-200b~200a~429 reporters, pcDNA3.1, pcDNA3.1 Zeb1, pcDNA3.1 Zeb2 or the pcDNA3.1 *miR-200b* *eRNA* plasmids, and the Renilla vector were co-transfected into epithelial MDA-MB-468 (white bars) or mesenchymal MDA-MB 231 (black bars) cells. Transiently transfected cells were incubated for 24-48 hours. Data are normalized by Renilla luciferase activity and are means ± SD of at least three independent experiments.
(TIF)

Figure S10 **Expression levels of EMT-affiliated genes in HMLE cells transfected with the pcDNA *miR-200b eRNA* plasmid.** (A) Relative expression levels of the (A) E-cadherin, N-cadherin, Zeb-1, Zeb-2, Twist and Fibronectin or (B) miR-200b, miR-200a and miR-429 in HMLE cells transfected with pcDNA control vector or the pcDNA *miR-200b* *eRNA* overexpression vector. Following DNaseI treatment, the RNA was subjected to cDNA synthesis using random hexamers. Data was analyzed using the comparative quantitation method and is shown as relative expression to pcDNA control (set to 1). GAPDH was used for normalization, and error bars represent mean ± SD of two independent experiments.
(TIF)

Table S1 **List of primers used for mRNA qPCR assays.**
(DOC)

Table S2 **List of primers used for eRNA qPCR mapping across the miR-200b~a~429 locus.**
(DOC)

Table S3 **List of primers used for miRNA qPCR assays.**
(DOC)

Table S4 **List of primers used for RACE-seq to determine the 5’ and 3’ ends of the miR200b eRNA transcript.**
(DOC)

Table S5 **List of primers used for ChIP–qPCR assays to tile the region of -10kb to +8kb from the TSS of the miR-200b~a~429 gene and control genes.**
(DOC)

## References

[1] Gangaraju VK, Lin H (2009) MicroRNAs: key regulators of stem cells. Nat Rev Mol Cell Biol 10: 116–125. doi:10.1038/nrm2621. PubMed: 19165214. 19165214 10.1038/nrm2621 PMC4118578

[2] Mani SA, Guo W, Liao MJ, Eaton EN, Ayyanan A et al. (2008) The epithelial-mesenchymal transition generates cells with properties of stem cells. Cell 133: 704–715. doi:10.1016/j.cell.2008.03.027. PubMed: 18485877. 18485877 10.1016/j.cell.2008.03.027 PMC2728032

[3] Brabletz S, Brabletz T (2010) The ZEB/miR-200 feedback loop--a motor of cellular plasticity in development and cancer? EMBO Rep 11: 670–677. doi:10.1038/embor.2010.117. PubMed: 20706219. 20706219 10.1038/embor.2010.117 PMC2933868

[4] Bracken CP, Gregory PA, Kolesnikoff N, Bert AG, Wang J et al. (2008) A double-negative feedback loop between ZEB1-SIP1 and the microRNA-200 family regulates epithelial-mesenchymal transition. Cancer Res 68: 7846–7854. doi:10.1158/0008-5472.CAN-08-1942. PubMed: 18829540. 18829540 10.1158/0008-5472.CAN-08-1942

[5] Gregory PA, Bert AG, Paterson EL, Barry SC, Tsykin A et al. (2008) The miR-200 family and miR-205 regulate epithelial to mesenchymal transition by targeting ZEB1 and SIP1. Nat Cell Biol 10: 593–601. doi:10.1038/ncb1722. PubMed: 18376396. 18376396 10.1038/ncb1722

[6] Hurteau GJ, Carlson JA, Spivack SD, Brock GJ (2007) Overexpression of the microRNA hsa-miR-200c leads to reduced expression of transcription factor 8 and increased expression of E-cadherin. Cancer Res 67: 7972–7976. doi:10.1158/0008-5472.CAN-07-1058. PubMed: 17804704. 17804704 10.1158/0008-5472.CAN-07-1058

[7] Burk U, Schubert J, Wellner U, Schmalhofer O, Vincan E et al. (2008) A reciprocal repression between ZEB1 and members of the miR-200 family promotes EMT and invasion in cancer cells. EMBO Rep 9: 582–589. doi:10.1038/embor.2008.74. PubMed: 18483486. 18483486 10.1038/embor.2008.74 PMC2396950

[8] Park SM, Gaur AB, Lengyel E, Peter ME (2008) The miR-200 family determines the epithelial phenotype of cancer cells by targeting the E-cadherin repressors ZEB1 and ZEB2. Genes Dev 22: 894–907. doi:10.1101/gad.1640608. PubMed: 18381893. 18381893 10.1101/gad.1640608 PMC2279201

[9] Korpal M, Lee ES, Hu G, Kang Y (2008) The miR-200 family inhibits epithelial-mesenchymal transition and cancer cell migration by direct targeting of E-cadherin transcriptional repressors ZEB1 and ZEB2. J Biol Chem 283: 14910–14914. doi:10.1074/jbc.C800074200. PubMed: 18411277. 18411277 10.1074/jbc.C800074200 PMC3258899

[10] Bulger M, Groudine M (2011) Functional and mechanistic diversity of distal transcription enhancers. Cell 144: 327–339. doi:10.1016/j.cell.2011.01.024. PubMed: 21295696. 21295696 10.1016/j.cell.2011.01.024 PMC3742076

[11] Jin F, Li Y, Ren B, Natarajan R (2011) Enhancers: multi-dimensional signal integrators. Transcription 2: 226–230. doi:10.4161/trns.2.5.17712. PubMed: 22231119. 22231119 10.4161/trns.2.5.17712 PMC3265780

[12] Deaton AM, Bird A (2011) CpG islands and the regulation of transcription. Genes Dev 25: 1010–1022. doi:10.1101/gad.2037511. PubMed: 21576262. 21576262 10.1101/gad.2037511 PMC3093116

[13] Van Bortle K, Corces VG (2012) Nuclear organization and genome function. Annu Rev Cell Dev Biol 28: 163–187. doi:10.1146/annurev-cellbio-101011-155824. PubMed: 22905954. 22905954 10.1146/annurev-cellbio-101011-155824 PMC3717390

[14] Pike JW (2011) Genome-scale techniques highlight the epigenome and redefine fundamental principles of gene regulation. Journal of bone and mineral research : the official journal of the American Society for Bone and Mineral Research 26: 1155–1162. doi:10.1002/jbmr.317. PubMed: 21611959. 10.1002/jbmr.317 PMC3312753 21611959

[15] Smallwood A, Ren B (2013) Genome organization and long-range regulation of gene expression by enhancers. Curr Opin Cell Biol, 25: 1–8. doi:10.1016/j.ceb.2013.02.005. 23465541 10.1016/j.ceb.2013.02.005 PMC4180870

[16] Heintzman ND, Stuart RK, Hon G, Fu Y, Ching CW et al. (2007) Distinct and predictive chromatin signatures of transcriptional promoters and enhancers in the human genome. Nat Genet 39: 311–318. doi:10.1038/ng1966. PubMed: 17277777. 17277777 10.1038/ng1966

[17] Heintzman ND, Ren B (2009) Finding distal regulatory elements in the human genome. Curr Opin Genet Dev 19: 541–549. doi:10.1016/j.gde.2009.09.006. PubMed: 19854636. 19854636 10.1016/j.gde.2009.09.006 PMC3321269

[18] Heintzman ND, Hon GC, Hawkins RD, Kheradpour P, Stark A et al. (2009) Histone modifications at human enhancers reflect global cell-type-specific gene expression. Nature 459: 108–112. doi:10.1038/nature07829. PubMed: 19295514. 19295514 10.1038/nature07829 PMC2910248

[19] Visel A, Blow MJ, Li Z, Zhang T, Akiyama JA et al. (2009) ChIP-seq accurately predicts tissue-specific activity of enhancers. Nature 457: 854–858. doi:10.1038/nature07730. PubMed: 19212405. 19212405 10.1038/nature07730 PMC2745234

[20] Creyghton MP, Cheng AW, Welstead GG, Kooistra T, Carey BW et al. (2010) Histone H3K27ac separates active from poised enhancers and predicts developmental state. Proc Natl Acad Sci U S A 107: 21931–21936. doi:10.1073/pnas.1016071107. PubMed: 21106759. 21106759 10.1073/pnas.1016071107 PMC3003124

[21] De Santa F, Barozzi I, Mietton F, Ghisletti S, Polletti S et al. (2010) A large fraction of extragenic RNA pol II transcription sites overlap enhancers. PLOS Biol 8: e1000384. doi:10.1371/journal.pbio.1000384. 20485488 10.1371/journal.pbio.1000384 PMC2867938

[22] Blow MJ, McCulley DJ, Li Z, Zhang T, Akiyama JA et al. (2010) ChIP-Seq identification of weakly conserved heart enhancers. Nat Genet 42: 806–810. doi:10.1038/ng.650. PubMed: 20729851. 20729851 10.1038/ng.650 PMC3138496

[23] Rada-Iglesias A, Bajpai R, Swigut T, Brugmann SA, Flynn RA et al. (2011) A unique chromatin signature uncovers early developmental enhancers in humans. Nature 470: 279–283. doi:10.1038/nature09692. PubMed: 21160473. 21160473 10.1038/nature09692 PMC4445674

[24] Pekowska A, Benoukraf T, Zacarias-Cabeza J, Belhocine M, Koch F et al. (2011) H3K4 tri-methylation provides an epigenetic signature of active enhancers. EMBO J 30: 4198–4210. doi:10.1038/emboj.2011.295. PubMed: 21847099. 21847099 10.1038/emboj.2011.295 PMC3199384

[25] Guttman M, Donaghey J, Carey BW, Garber M, Grenier JK et al. (2011) lincRNAs act in the circuitry controlling pluripotency and differentiation. Nature 477: 295–300. doi:10.1038/nature10398. PubMed: 21874018. 21874018 10.1038/nature10398 PMC3175327

[26] Ørom UA, Shiekhattar R (2011) Long non-coding RNAs and enhancers. Curr Opin Genet Dev 21: 194–198. doi:10.1016/j.gde.2011.01.020. PubMed: 21330130. 21330130 10.1016/j.gde.2011.01.020 PMC3779066

[27] Kim TK, Hemberg M, Gray JM, Costa AM, Bear DM et al. (2010) Widespread transcription at neuronal activity-regulated enhancers. Nature 465: 182–187. doi:10.1038/nature09033. PubMed: 20393465. 20393465 10.1038/nature09033 PMC3020079

[28] Melo CA, Drost J, Wijchers PJ, Van de Werken H, De Wit E et al. (2013) eRNAs Are Required for p53-Dependent Enhancer Activity and Gene Transcription. Mol Cell 49: 524–535. doi:10.1016/j.molcel.2012.11.021. PubMed: 23273978. 23273978 10.1016/j.molcel.2012.11.021

[29] Flynn RA, Chang HY (2012) Active chromatin and noncoding RNAs: an intimate relationship. Curr Opin Genet Dev 22: 172–178. doi:10.1016/j.gde.2011.11.002. PubMed: 22154525. 22154525 10.1016/j.gde.2011.11.002 PMC3319162

[30] Kowalczyk MS, Hughes JR, Garrick D, Lynch MD, Sharpe JA et al. (2012) Intragenic enhancers act as alternative promoters. Mol Cell 45: 447–458. doi:10.1016/j.molcel.2011.12.021. PubMed: 22264824. 22264824 10.1016/j.molcel.2011.12.021

[31] Zentner GE, Scacheri PC (2012) The chromatin fingerprint of gene enhancer elements. J Biol Chem 287: 30888–30896. doi:10.1074/jbc.R111.296491. PubMed: 22952241. 22952241 10.1074/jbc.R111.296491 PMC3438921

[32] Ebisuya M, Yamamoto T, Nakajima M, Nishida E (2008) Ripples from neighbouring transcription. Nat Cell Biol 10: 1106–1113. doi:10.1038/ncb1771. PubMed: 19160492. 19160492 10.1038/ncb1771

[33] Iliopoulos D, Lindahl-Allen M, Polytarchou C, Hirsch HA, Tsichlis PN et al. (2010) Loss of miR-200 inhibition of Suz12 leads to polycomb-mediated repression required for the formation and maintenance of cancer stem cells. Mol Cell 39: 761–772. doi:10.1016/j.molcel.2010.08.013. PubMed: 20832727. 20832727 10.1016/j.molcel.2010.08.013 PMC2938080

[34] Vrba L, Garbe JC, Stampfer MR, Futscher BW (2011) Epigenetic regulation of normal human mammary cell type-specific miRNAs. Genome Res 21: 2026–2037. doi:10.1101/gr.123935.111. PubMed: 21873453. 21873453 10.1101/gr.123935.111 PMC3227093

[35] Cao Q, Mani RS, Ateeq B, Dhanasekaran SM, Asangani IA, et al. (2011) Coordinated regulation of polycomb group complexes through microRNAs in cancer. Cancer cell 20: 187–199. doi:10.1016/j.ccr.2011.06.016. 21840484 10.1016/j.ccr.2011.06.016 PMC3157014

[36] Lim YY, Wright JA, Attema JA, Gregory PA, Bert AG, et al. (n.d.) Epigenetic modulation of the miR-200 family is associated with transition to a breast cancer stem cell-like state. Journal of cell science 126:2256-66. 10.1242/jcs.122275 23525011

[37] Elenbaas B (2001) Human breast cancer cells generated by oncogenic transformation of primary mammary epithelial cells. Genes Dev 15: 50–65. doi:10.1101/gad.828901. PubMed: 11156605. 11156605 10.1101/gad.828901 PMC312602

[38] Gregory PA, Bracken CP, Bert AG, Goodall GJ (2008) MicroRNAs as regulators of epithelial-mesenchymal transition. Cell Cycle 7: 3112–3118. doi:10.4161/cc.7.20.6851. PubMed: 18927505. 18927505 10.4161/cc.7.20.6851

[39] Himes SR, Tagoh H, Goonetilleke N, Sasmono T, Oceandy D et al. (2001) A highly conserved c-fms gene intronic element controls macrophage-specific and regulated expression. J Leukoc Biol 70: 812–820. PubMed: 11698502. 11698502

[40] Lefevre P, Witham J, Lacroix CE, Cockerill PN, Bonifer C (2008) The LPS-induced transcriptional upregulation of the chicken lysozyme locus involves CTCF eviction and noncoding RNA transcription. Mol Cell 32: 129–139. doi:10.1016/j.molcel.2008.07.023. PubMed: 18851839. 18851839 10.1016/j.molcel.2008.07.023 PMC2581490

[41] Ørom UA, Derrien T, Beringer M, Gumireddy K, Gardini A et al. (2010) Long noncoding RNAs with enhancer-like function in human cells. Cell 143: 46–58. doi:10.1016/j.cell.2010.09.001. PubMed: 20887892. 20887892 10.1016/j.cell.2010.09.001 PMC4108080

[42] Kanhere A, Viiri K, Araújo CC, Rasaiyaah J, Bouwman RD et al. (2010) Short RNAs are transcribed from repressed polycomb target genes and interact with polycomb repressive complex-2. Mol Cell 38: 675–688. doi:10.1016/j.molcel.2010.03.019. PubMed: 20542000. 20542000 10.1016/j.molcel.2010.03.019 PMC2886029

[43] Sandelin A, Carninci P, Lenhard B, Ponjavic J, Hayashizaki Y et al. (2007) Mammalian RNA polymerase II core promoters: insights from genome-wide studies. Nat Rev Genet 8: 424–436. doi:10.1038/nrg2026. PubMed: 17486122. 17486122 10.1038/nrg2026

[44] Frith MC, Valen E, Krogh A, Hayashizaki Y, Carninci P et al. (2008) A code for transcription initiation in mammalian genomes. Genome Res 18: 1–12. doi:10.1101/gr.6831208. PubMed: 18032727. 18032727 10.1101/gr.6831208 PMC2134772

[45] Boussouar F, Rousseaux S, Khochbin S (2008) A new insight into male genome reprogramming by histone variants and histone code. Cell Cycle 7: 3499–3502. doi:10.4161/cc.7.22.6975. PubMed: 19001855. 19001855 10.4161/cc.7.22.6975

[46] Weake VM, Workman JL (2010) Inducible gene expression: diverse regulatory mechanisms. Nat Rev Genet 11: 426–437. doi:10.1038/nrg2781. PubMed: 20421872. 20421872 10.1038/nrg2781

[47] Calo E, Wysocka J (2013) Modification of Enhancer Chromatin: What, How, and Why? Mol Cell 49: 825–837. doi:10.1016/j.molcel.2013.01.038. PubMed: 23473601. 23473601 10.1016/j.molcel.2013.01.038 PMC3857148

[48] Wang KC, Yang YW, Liu B, Sanyal A, Corces-Zimmerman R et al. (2011) A long noncoding RNA maintains active chromatin to coordinate homeotic gene expression. Nature 472: 120–124. doi:10.1038/nature09819. PubMed: 21423168. 21423168 10.1038/nature09819 PMC3670758

[49] Bertani S, Sauer S, Bolotin E, Sauer F (2011) The noncoding RNA Mistral activates Hoxa6 and Hoxa7 expression and stem cell differentiation by recruiting MLL1 to chromatin. Mol Cell 43: 1040–1046. doi:10.1016/j.molcel.2011.08.019. PubMed: 21925392. 21925392 10.1016/j.molcel.2011.08.019 PMC3176448

