# Supplementary figures and images for: Identification of an Enhancer That Increases miR-200b~200a~429 Gene Expression in Breast Cancer Cells
Source: PLoS One. 2013 Sep 25;8(9):e75517. doi: 10.1371/journal.pone.0075517 (PMC3783398; doi:10.1371/journal.pone.0075517)

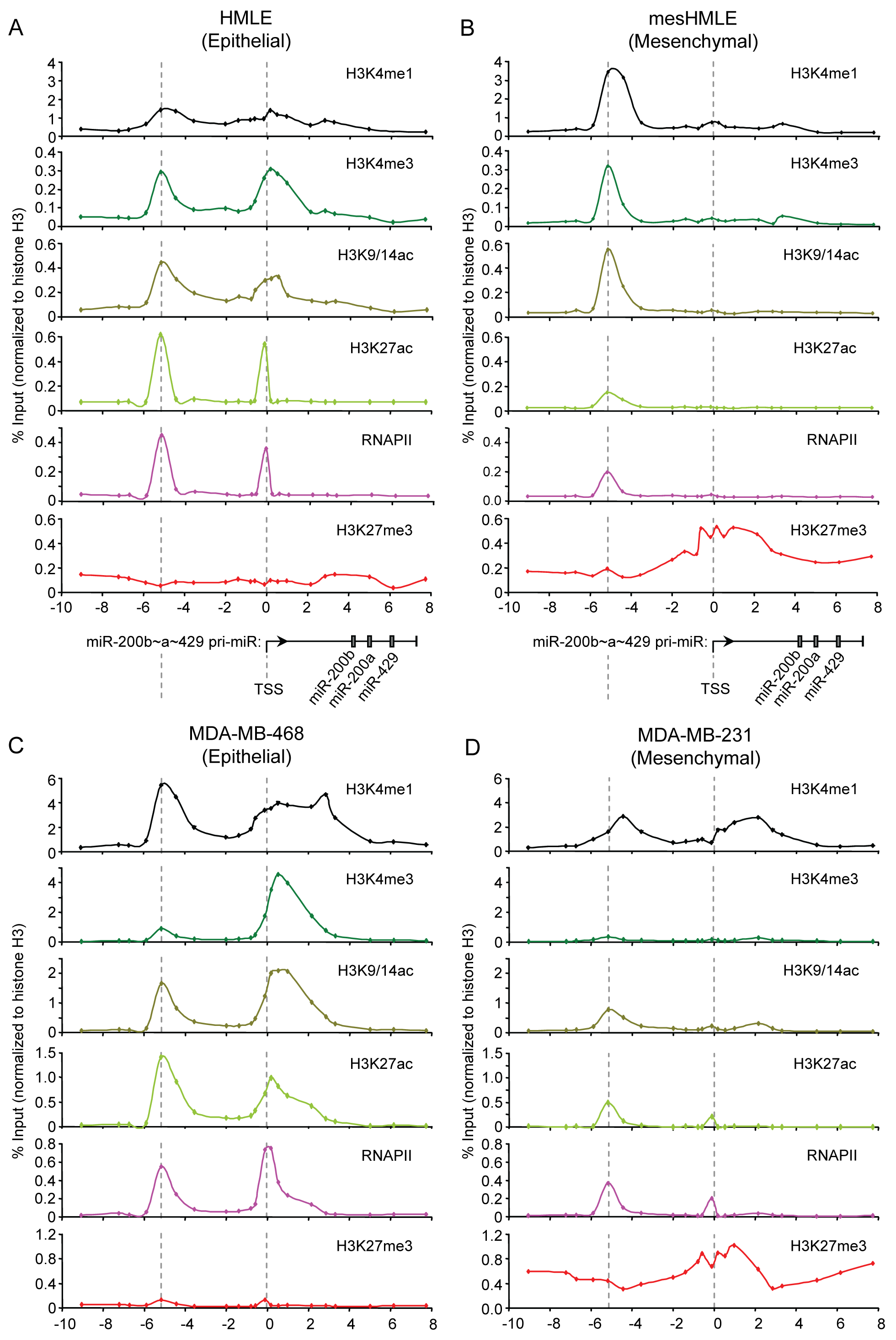

Supplement: Figure S1 — An active chromatin domain upstream of the miR-200b~200a~429 locus confirmed by ChIP-qPCR analysis. Representative ChIP-qPCR analysis of H3K4me1, H3K4me3, H3K9/14ac, H3K27ac and H3K27me3 at the miR-200b~200a~429 locus (chr1:1,090,000 -1,105,000) in (A) epithelial HMLE, (B) mesHMLE, (C) MDA-MB-468 and (D) MDA-MB-231. The x-axis shows the distance up and downstream relative to the TSS and a schematic diagram of the primary miR-200b~200a~429 transcript is positioned to scale. An arrow marks the TSS and shaded boxes indicates the mature miRNA transcripts. The y-axis shows % Input of histone H3 modifications normalized to unmodified histone H3. (TIF) [file pone.0075517.s001.tif]

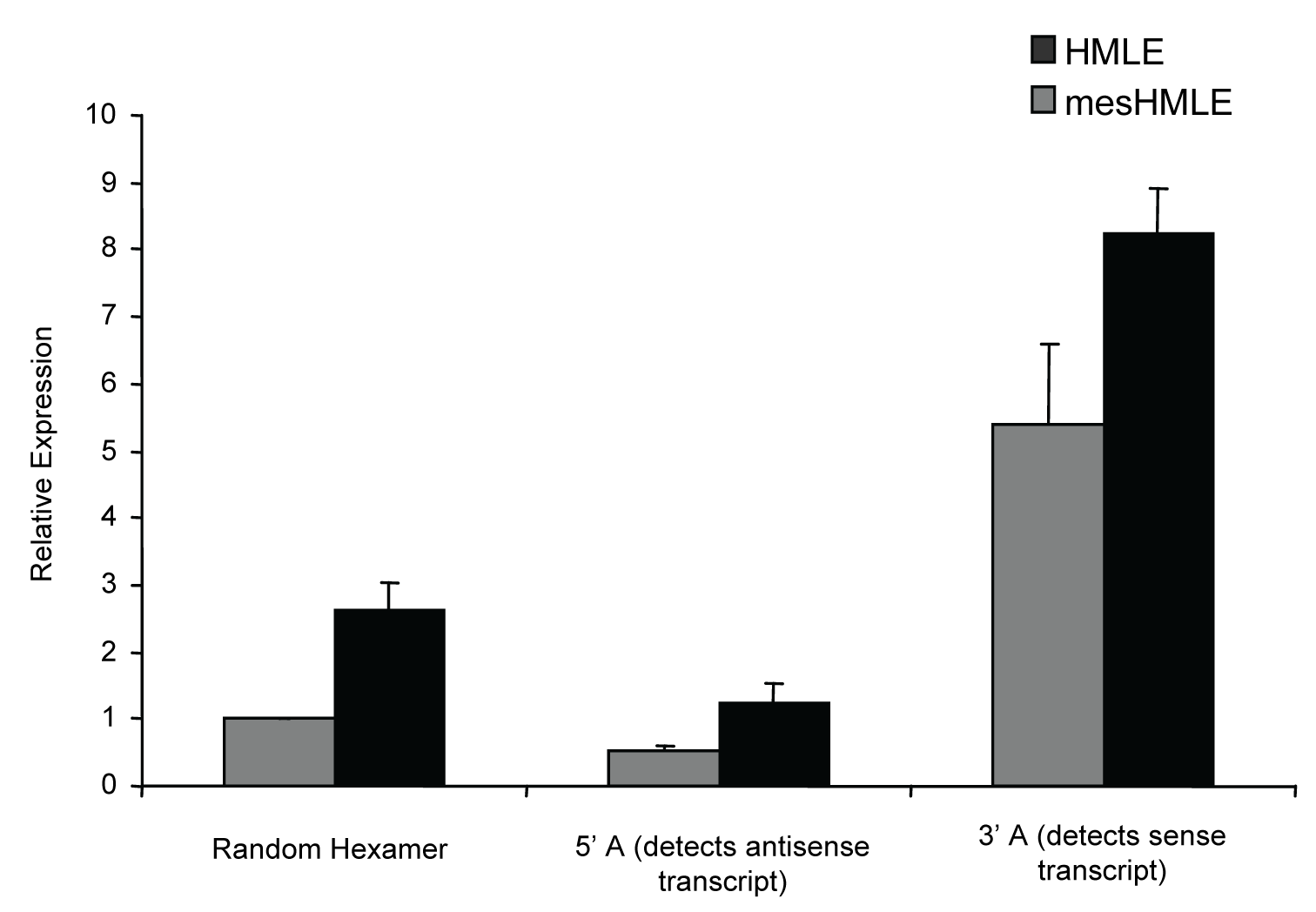

Supplement: Figure S2 — The miR-200b~200a~429 enhancer region produces sense and antisense eRNA transcripts. Total RNA was isolated from HMLE and mesHMLE cells. Following DNaseI treatment, total RNA was converted to cDNA using random hexamers, 5’ A RACE primer (complementary to the sense RNA transcript) or 3’ A RACE primer (complementary to the antisense RNA transcript) (Table S4). Real-time PCR analysis of cDNA was performed using gene specific primers for the eRNA (Table S1). GAPDH was used for normalization and data was analyzed using the comparative quantitation method shown as relative expression to HMLE random hexamer primed cDNA (set to 1). Error bars represent mean ± SD of two independent experiments. (TIF) [file pone.0075517.s002.tif]

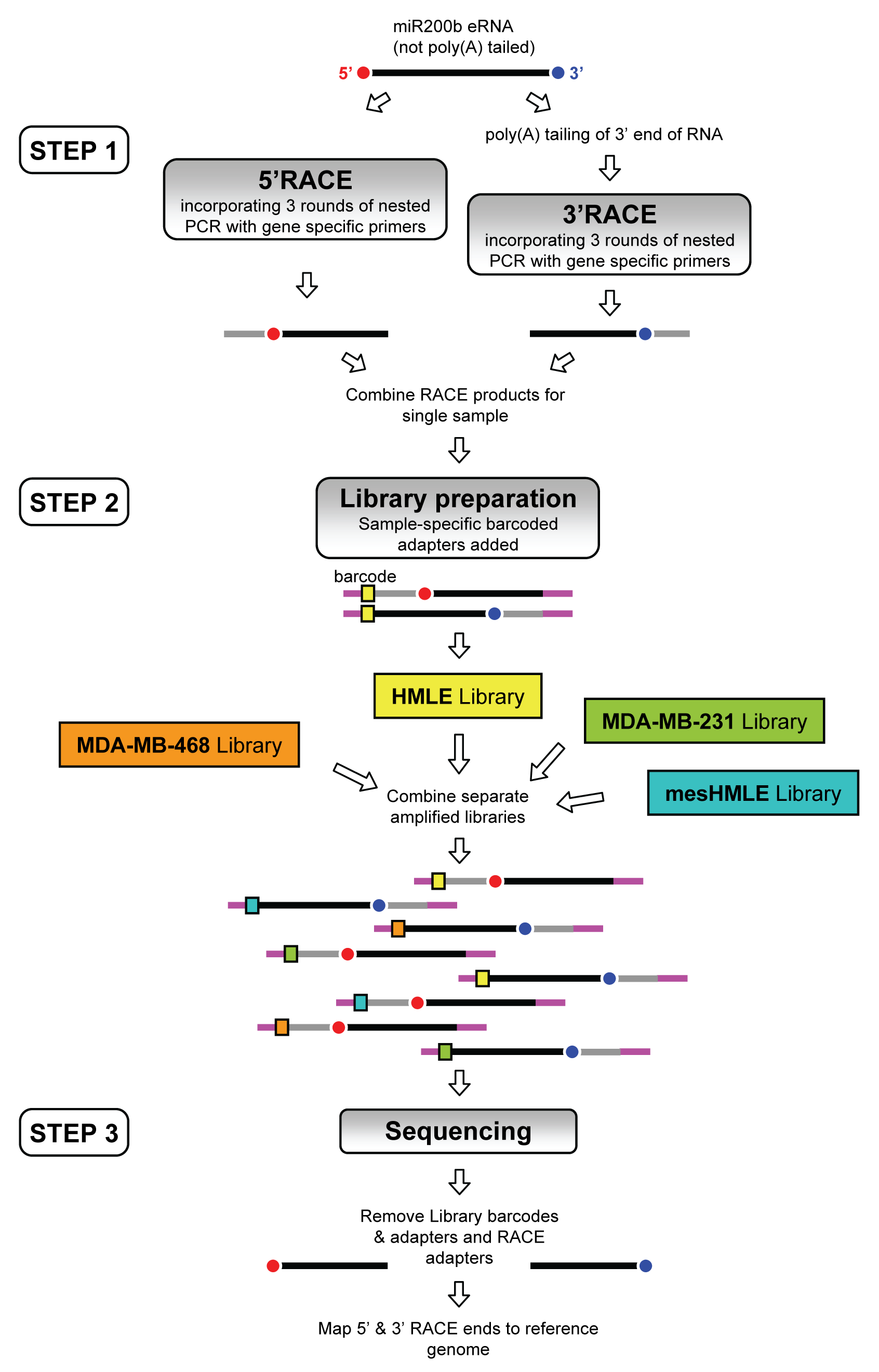

Supplement: Figure S3 — Schematic of the 5’ and 3’ RACE-seq methodology. The RACE-seq method comprises three steps, 5’ and 3’ RACE, Library preparation and Sequencing. DNaseI-treated total RNA isolated from HMLE, mesHMLE, MDA-MB-231 and MDA-MB-468 was subjected to 5’ RACE by incorporating three rounds of nested PCR using gene specific primers (Table S3) (Step 1). 3’ RACE was performed in a similar manner except that the DNaseI-treated total RNA was first polyA tailed (E. coli Poly(A) Polymerase I) (Step 1). 5’ and 3’ RACE PCR products obtained from each cell type were pooled into single reaction tube and subjected to library preparation (Step 2). Individual libraries comprising RACE products from each cell line (total of 4) were prepared using sample-specific bar code adapters were then combined and sequenced together (Step 3). Sequences obtained from each cell type were identified using their unique bar codes. For each sequencing read, the bar codes were read, trimmed and sorted into 4 bins (corresponding to each cell line RACE pool). The Ion Torrent library preparation adapters (pink bars) and the poly(n) sequences and adapters added during the RACE protocol (grey bars) were removed, leaving behind specific sequences corresponding to either 5’ or 3’ RACE products (black bars with either a red dot or blue dot representing the respective transcript ends). These sequences were mapped to the human hg19 reference genome and 5’ or 3’ ends were identified. (TIF) [file pone.0075517.s003.tif]

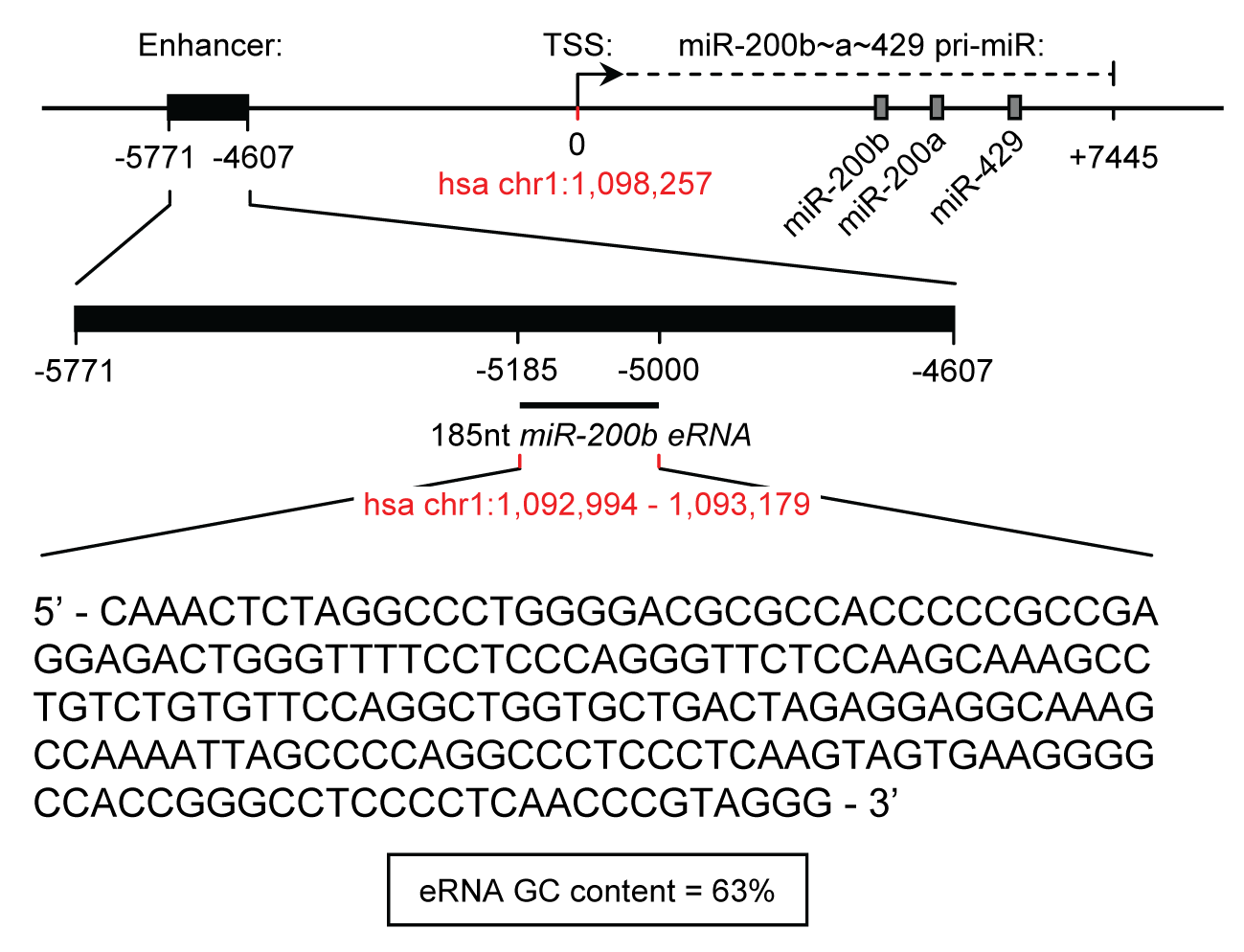

Supplement: Figure S4 — Schematic of the miR-200b eRNA transcript and its genomic location on human chromosome 1. The major 5’ and 3’ RACE-seq transcript occurring in epithelial HMLE and mesHMLE cells is shown inset to the location of the transcript produced at enhancer region on human chromosome 1 (hsa chr1:1,092,994-1,093,179). The GC content is indicated. (TIF) [file pone.0075517.s004.tif]

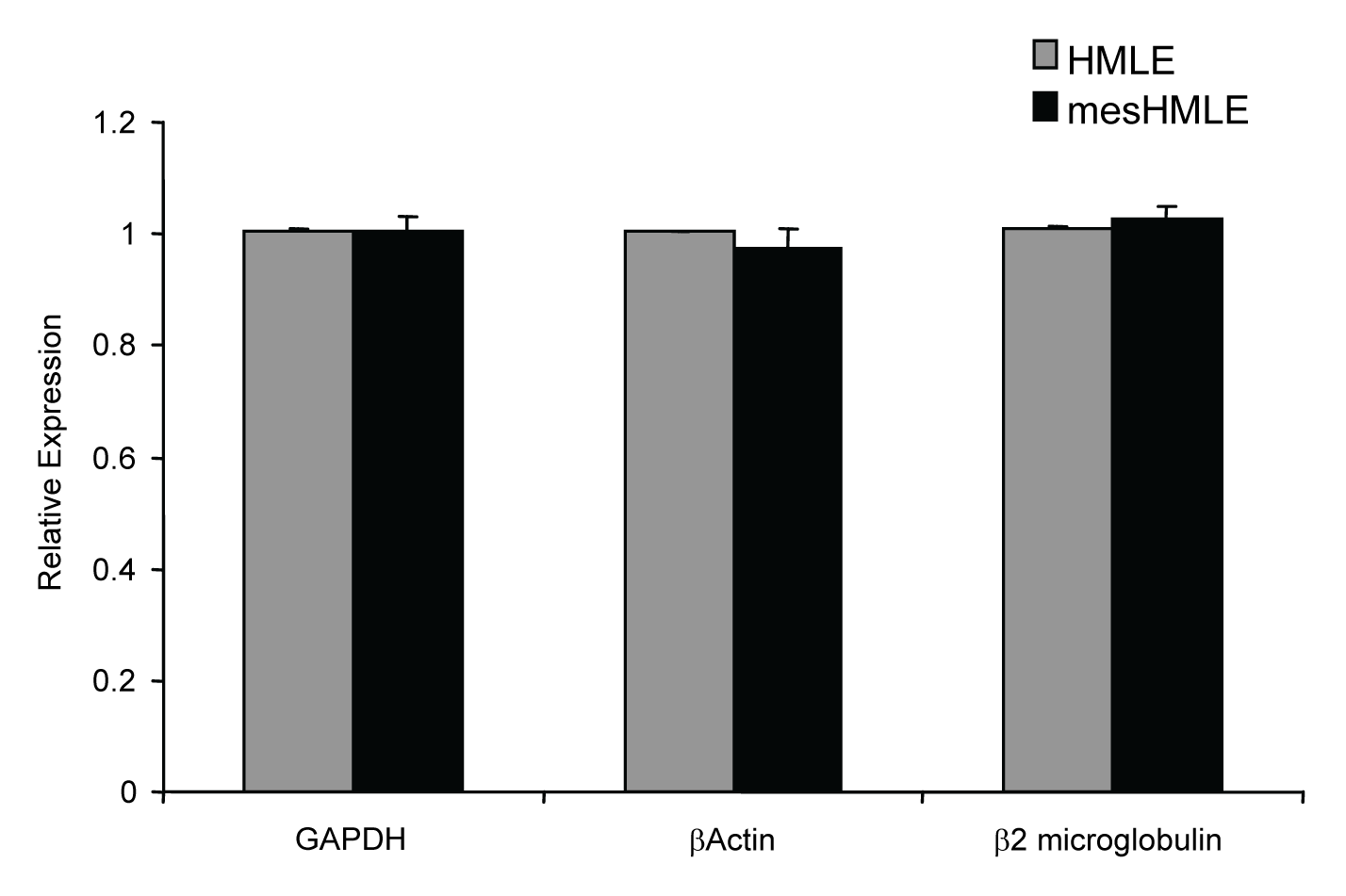

Supplement: Figure S5 — GAPDH is suitable for use as a normalization control gene in the HMLE EMT cell line model. Relative expression levels of the housekeeping genes GAPDH, βActin and β2-microglobulin in the HMLE and mesHMLE cells. Following DNaseI treatment, the RNA was converted to cDNA using random hexamers. Real-time PCR analysis of cDNA was performed using gene specific primers. The data was analyzed using the comparative quantitation method and is shown as relative expression to HMLE (set to 1) for each mRNA tested. Error bars represent mean ± SD of two independent experiments. (TIF) [file pone.0075517.s005.tif]

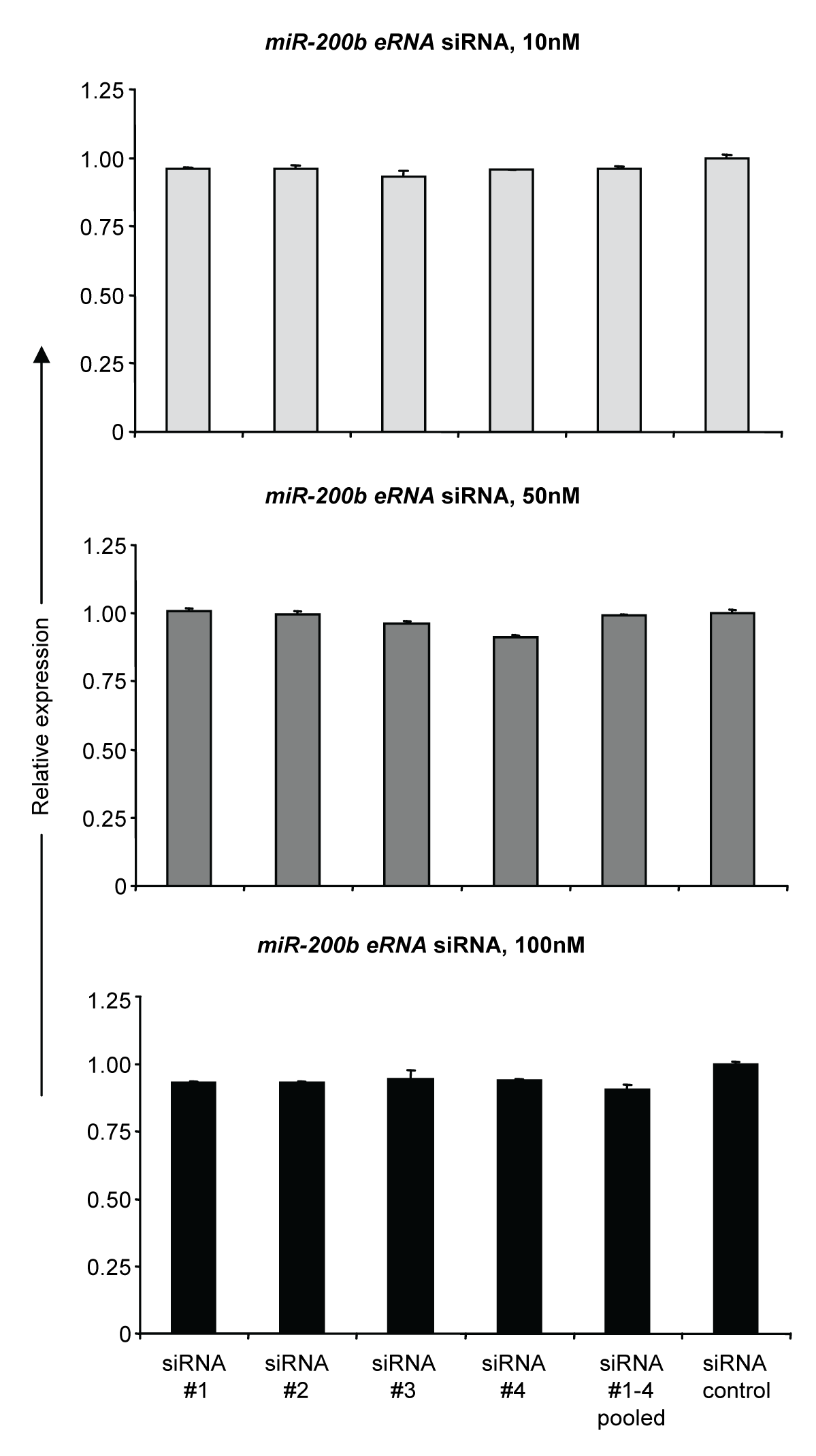

Supplement: Figure S6 — Custom designed siRNAs fail to knock down miR-200b eRNA transcript. Four custom siRNAs were tested in transient transfection assays for their ability to knockdown miR-200b eRNA in HMLE cells. Individual and pooled siRNAs (1-4) were assayed at 10 nM (top panel), 50 nM (middle panel) and 100 nM (bottom panel). Following DNaseI treatment, the RNA was converted to cDNA using random hexamers. Real-time PCR analysis of cDNA was performed using gene specific primers for miR-200b eRNA (Table S1). Quantitative RT-PCR data is calculated using the comparative quantitation method and is shown as relative expression to the control siRNA (set to 1) following GAPDH normalization. Error bars represent mean ± SD of three independent experiments. (TIF) [file pone.0075517.s006.tif]

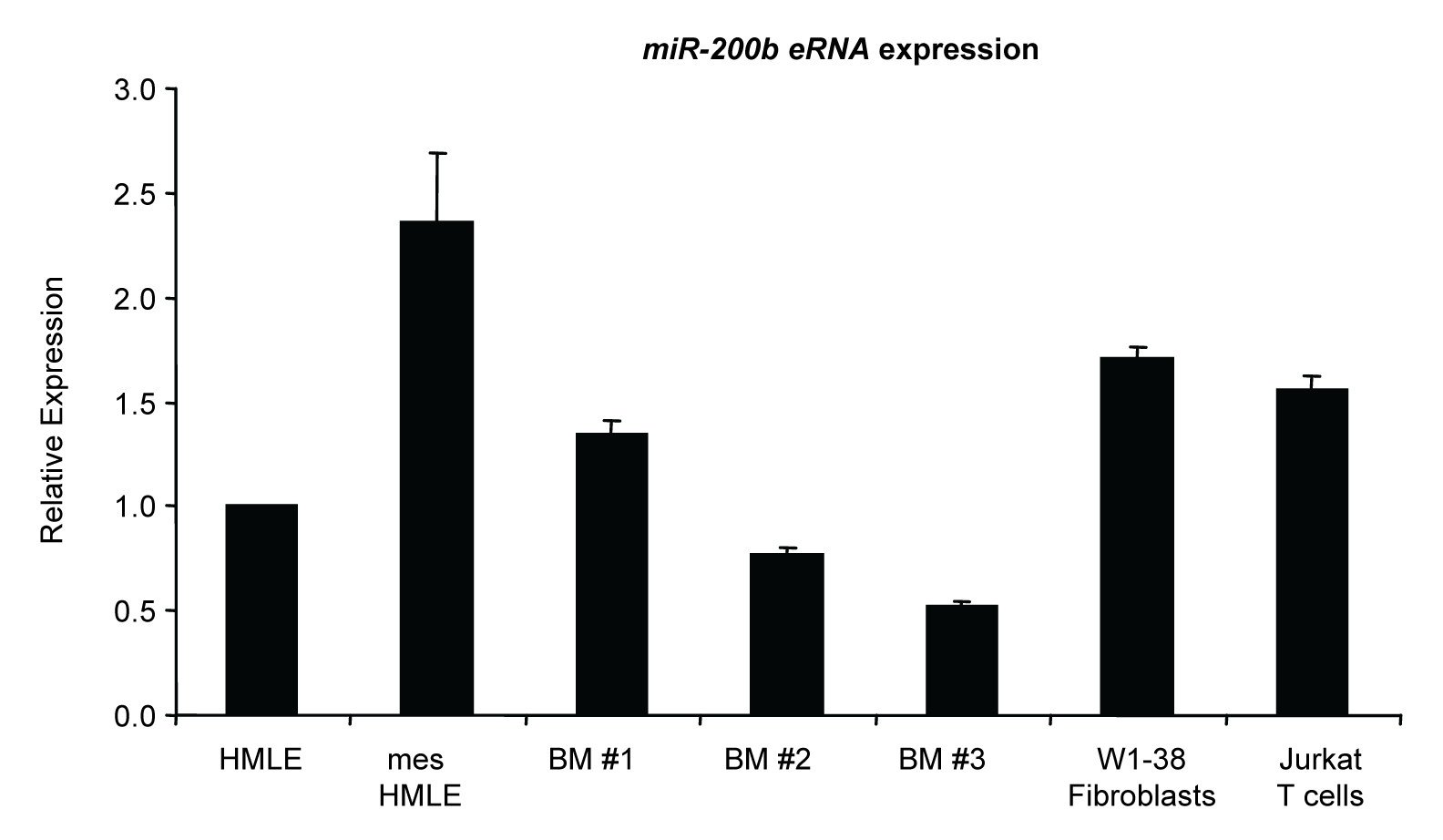

Supplement: Figure S7 — Gene expression analysis of miR-200b eRNA in other cell types. Total RNA was isolated from HMLE, mesHMLE, normal bone marrow cells (samples 1-3), W1-38 fibroblast cell line and the Jurkat T cell line. Following DNaseI treatment, the RNA was converted to cDNA using random hexamers. Real-time PCR analysis of cDNA was performed using gene specific primers for miR-200b eRNA (Table S1). GAPDH was used for normalization. Data was analyzed using the comparative quantitation method and is shown as relative expression to HMLE (set to 1). Error bars represent mean ± SD of three independent experiments. (TIF) [file pone.0075517.s007.tif]

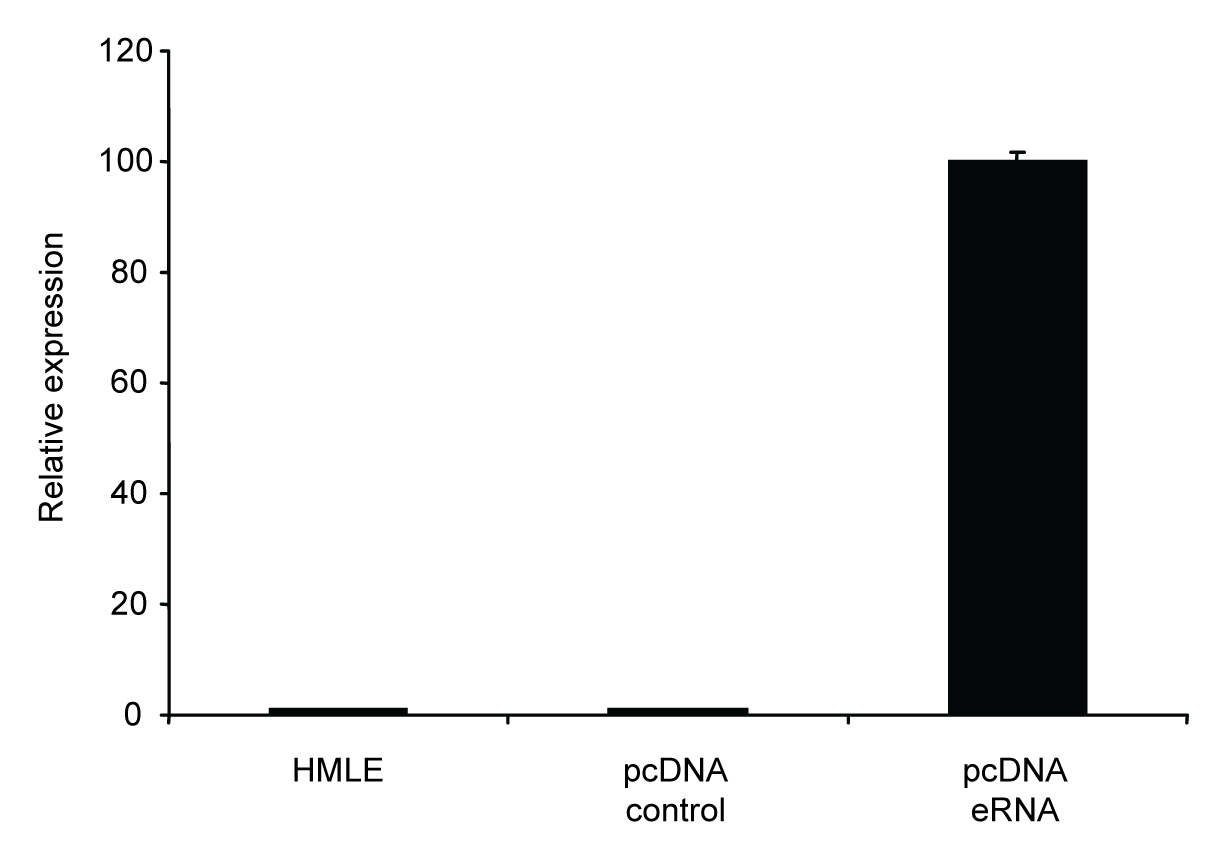

Supplement: Figure S8 — HMLE cells transiently transfected with the pcDNA miR-200b eRNA plasmid results in ~100 fold increased expression level. Relative expression using the comparative quantitation method of miR-200b eRNA in HMLE cells set to 1 (non-transfected), HMLE cells transiently transfected with the pcDNA control vector or the pcDNA miR-200b eRNA overexpression vector. GAPDH was used for normalization, and error bars represent mean ± SD of three independent experiments. (TIF) [file pone.0075517.s008.tif]

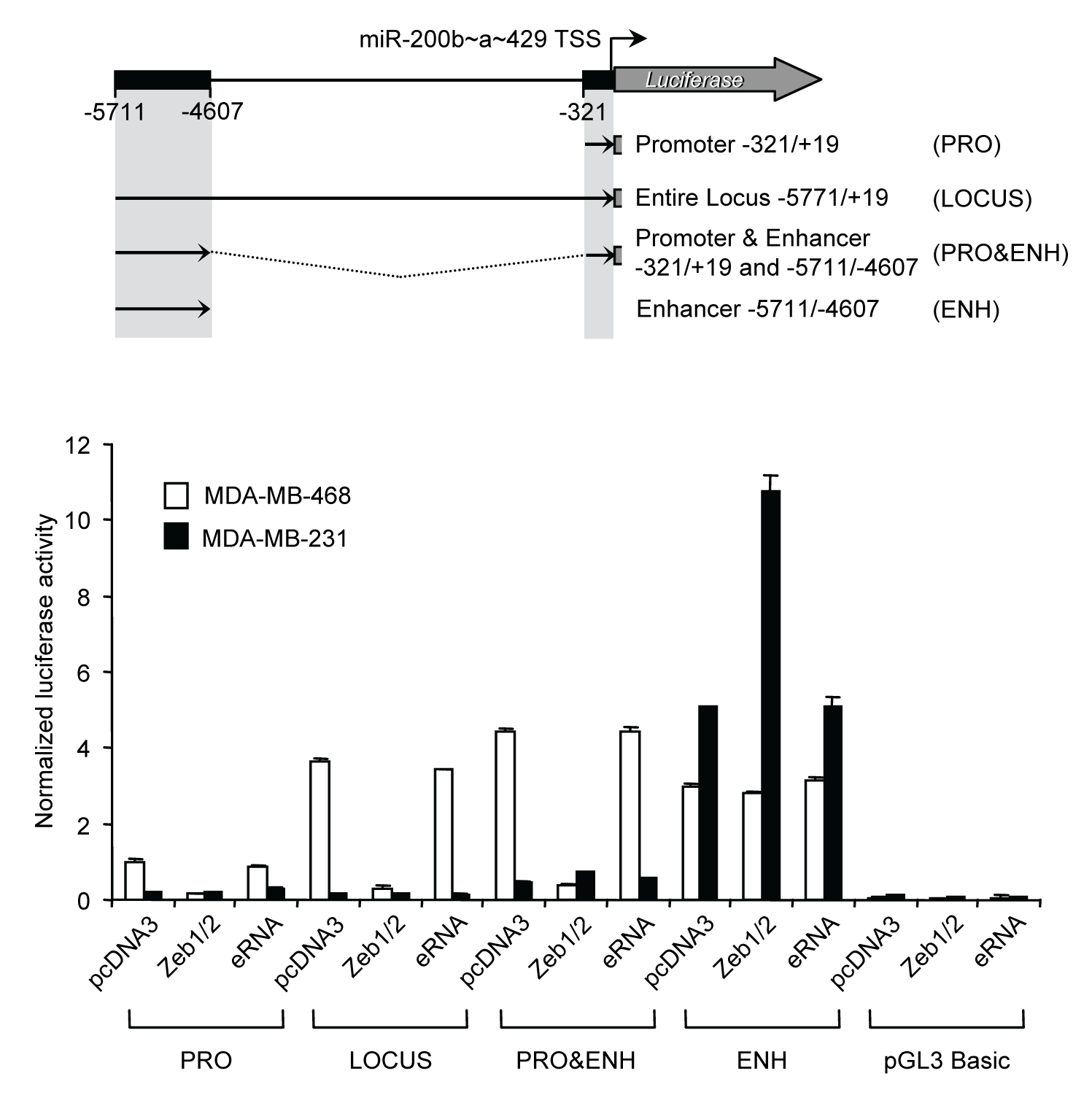

Supplement: Figure S9 — Over-expression of miR-200b eRNA has no effect on miR-200b~200a~429 promoter activity in MDA-MB-468 and MDA-MB-231 breast cancer cell lines. Schematic representation of the miR-200b~200a~429 reporter constructs, PRO (-321/+19), LOCUS (-5771/+19), PRO&ENH (-321/+19 to -5711/4607) and ENH (-5711/-4607). The miR-200b~200a~429 reporters, pcDNA3.1, pcDNA3.1 Zeb1, pcDNA3.1 Zeb2 or the pcDNA3.1 miR-200b eRNA plasmids, and the Renilla vector were co-transfected into epithelial MDA-MB-468 (white bars) or mesenchymal MDA-MB 231 (black bars) cells. Transiently transfected cells were incubated for 24-48 hours. Data are normalized by Renilla luciferase activity and are means ± SD of at least three independent experiments. (TIF) [file pone.0075517.s009.tif]

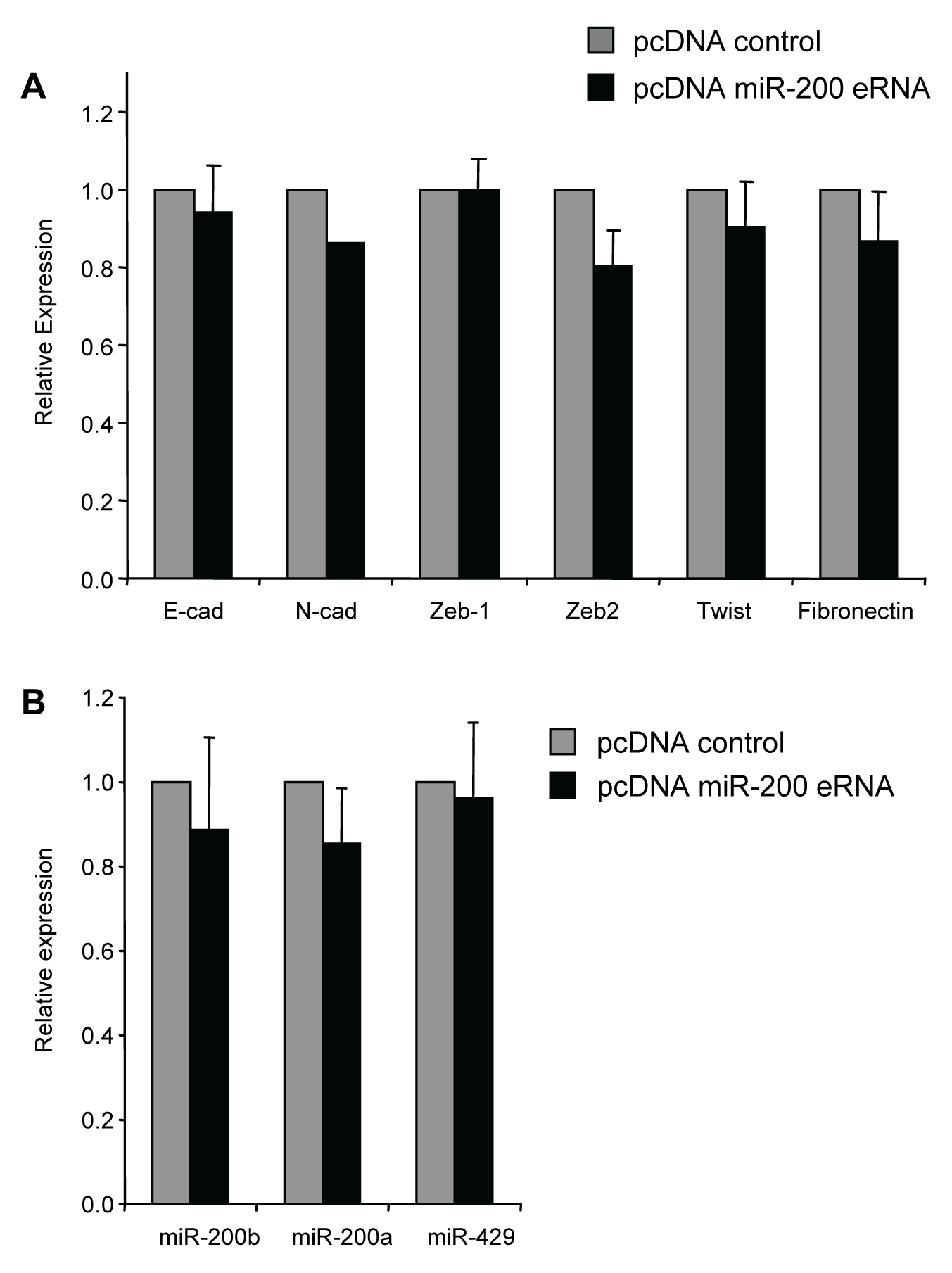

Supplement: Figure S10 — Expression levels of EMT-affiliated genes in HMLE cells transfected with the pcDNA miR-200b eRNA plasmid. (A) Relative expression levels of the (A) E-cadherin, N-cadherin, Zeb-1, Zeb-2, Twist and Fibronectin or (B) miR-200b, miR-200a and miR-429 in HMLE cells transfected with pcDNA control vector or the pcDNA miR-200b eRNA overexpression vector. Following DNaseI treatment, the RNA was subjected to cDNA synthesis using random hexamers. Data was analyzed using the comparative quantitation method and is shown as relative expression to pcDNA control (set to 1). GAPDH was used for normalization, and error bars represent mean ± SD of two independent experiments. (TIF) [file pone.0075517.s010.tif]
