# Supplementary material for: Identification of an Enhancer That Increases miR-200b~200a~429 Gene Expression in Breast Cancer Cells
Source: PLoS One. 2013 Sep 25;8(9):e75517. doi: 10.1371/journal.pone.0075517 (PMC3783398; doi:10.1371/journal.pone.0075517)
Supplement: Table S1 — List of primers used for mRNA qPCR assays. (DOC) [file pone.0075517.s011.doc]

**Table S1.** List of primers used for mRNA qPCR assays.

| **Gene** | **Forward (5’-3’)** | **Reverse (5’-3’)** |
| --- | --- | --- |
| GAPDH | ACCCAGAAGACTGTGGATGG | CAGTGAGCTTCCCGTTCAG |
| B-Actin | CCTGGCACCCAGCACAA | CTTGCGCTCAGGAGGAGC |
| β2-microglobulin | AGGCTATCCAGCGTACTCCA | TCAATGTCGGATGGATGAAA |
| E-cadherin | CCCACCACGTACAAGGGTC | CTGGGGTATTGGGGGCATC |
| Zeb1 | TTCAAACCCATAGTGGTTGCT | TGGGAGATACCAAACCAACTG |
| HOTAIR | GGTAGAAAAAGCAACCACGAAGC | ACATAAACCTCTGTCTGTGAGTGCC |
| 200b eRNA | gttttcctcccagggttctc | gtggccccttcactacttga |
| N-cadherin | CAACTTGCCAGAAAACTCCAGG | ATGAAACCGGGCTATCTGCTC |
| Twist | GTCCGCAGTCTTACGAGGAG | GCTTGAGGGTCTGAATCTTGCT |
| Fibronectin | CCATCAGCAGGAACACCTTT | TGGAGGTTAGTGGGAGCATC |
| Zeb2 | CAAGAGGCGCAAACAAGCC | GGTTGGCAATACCGTCATCC |
