# Supplementary material for: Identification of an Enhancer That Increases miR-200b~200a~429 Gene Expression in Breast Cancer Cells
Source: PLoS One. 2013 Sep 25;8(9):e75517. doi: 10.1371/journal.pone.0075517 (PMC3783398; doi:10.1371/journal.pone.0075517)
Supplement: Table S2 — List of primers used for eRNA qPCR mapping across the miR-200b~a~429 locus. (DOC) [file pone.0075517.s012.doc]

**Table S2.** List of primers used for eRNA qPCR mapping across the miR-200b~a~429 locus.

| **Genomic region** | **Forward (5’-3’)** | **Reverse (5’-3’)** |
| --- | --- | --- |
| -6.716kb | acacctgctcagctggaatc | gtctggaggctgggaagaat |
| -5.888kb | gtgaccttctggcttgaacc | accttttctcccactttgtcc |
| -5.379kb | CTCAGACGCTGTGCAGTGAG | CGAGGCTCTCGAGACAGAAC |
| -5.172kb | gttttcctcccagggttctc | gtggccccttcactacttga |
| -4.997kb | caagtagtgaaggggccac | GGCGGGATGGCTGGAGGCT |
| -4.02kb | agctgtgtccctgcctga | acttcctgatggcaccagac |
| -3.416kb | cagaaaactctggggccttt | acttagctggctgtggtggt |
| -3.264kb | GCACCTACCTGAGACCAAGG | CTGGCCTTAGACCCCAGAA |
| -3.07kb | GACCAGTTTCCAGCGAGAAG | ACCCCACGGTTTTCCATAAT |
| +2.148kb | TCGAAACTCTCCCAGAGACG | GACCTGCAAGGGTGAGCTT |
| +7.165kb | TGTGGTCAGCTCAGGTCAAG | GTAGCTCCCTTCAGCACAGG |

Genomic location is relative to the miR-200b~a~429 TSS
