# Supplementary material for: Identification of an Enhancer That Increases miR-200b~200a~429 Gene Expression in Breast Cancer Cells
Source: PLoS One. 2013 Sep 25;8(9):e75517. doi: 10.1371/journal.pone.0075517 (PMC3783398; doi:10.1371/journal.pone.0075517)
Supplement: Table S3 — List of primers used for miRNA qPCR assays. (DOC) [file pone.0075517.s013.doc]

**Table S3.** List of primers used for miRNA qPCR assays.

| **miRNA Taqman Assay** | **LifeTechnologies Assay ID** |
| --- | --- |
| hsa-miR-200b | 002251 |
| hsa-miR-200a | 000502 |
| hsa-miR-429 | 001024 |
| U6 snRNA | 001973 |
