# Supplementary material for: Identification of an Enhancer That Increases miR-200b~200a~429 Gene Expression in Breast Cancer Cells
Source: PLoS One. 2013 Sep 25;8(9):e75517. doi: 10.1371/journal.pone.0075517 (PMC3783398; doi:10.1371/journal.pone.0075517)
Supplement: Table S4 — List of primers used for RACE-seq to determine the 5’ and 3’ ends of the miR200b eRNA transcript. (DOC) [file pone.0075517.s014.doc]

**Table S4.** List of primers used for RACE-seq to determine the 5’ and 3’ ends of the miR200b eRNA transcript.

| **primer** | **Sequence (5’-3’)** |
| --- | --- |
| ***5’RACE*** | |
| 5’ A | gtggccccttcactacttga |
| 5’ B | TGGCTTTGCCTCCTCTAGTC |
| 5’ C | GCACCAGCCTGGAACACAGA |
| Abridged Anchor primer | 5’ RACE kit (Invitrogen) |
| AUAP | 5’ RACE kit (Invitrogen) |
| ***3’RACE*** | |
| Adapter primer | 3’RACE kit (Invitrogen) |
| 3’ A | cgaggagactgggttttcct |
| 3’ B | gactagaggaggcaaagcca |
| 3’ C | tcaagtagtgaaggggccac |
| Universal Amplification primer | 3’RACE kit (Invitrogen) |
| Abridged Universal Amplification primer | 3’RACE kit (Invitrogen) |
| ***RACE-seq library preparation*** | |
| Ion P1 Adapter | Ion Xpress Barcode Adapters 1–16 Kit (Life Technologies) |
| Ion Xpress Barcode 5 | Ion Xpress Barcode Adapters 1–16 Kit (Life Technologies) |
| Ion Xpress Barcode 6 | Ion Xpress Barcode Adapters 1–16 Kit (Life Technologies) |
| Ion Xpress Barcode 7 | Ion Xpress Barcode Adapters 1–16 Kit (Life Technologies) |
| Ion Xpress Barcode 8 | Ion Xpress Barcode Adapters 1–16 Kit (Life Technologies) |
