# Supplementary material for: Identification of an Enhancer That Increases miR-200b~200a~429 Gene Expression in Breast Cancer Cells
Source: PLoS One. 2013 Sep 25;8(9):e75517. doi: 10.1371/journal.pone.0075517 (PMC3783398; doi:10.1371/journal.pone.0075517)
Supplement: Table S5 — List of primers used for ChIP–qPCR assays to tile the region of -10kb to +8kb from the TSS of the miR-200b~a~429 gene and control genes. (DOC) [file pone.0075517.s015.doc]

**Table S5.** List of primers used for ChIP–qPCR assays to tile the region of -10 to +8kb from the TSS of the miR-200b~a~429 gene and control genes.

| **Genomic region** | **Forward (5’-3’)** | **Reverse (5’-3’)** |
| --- | --- | --- |
| -9.053kb | caactcatcccccagtcttg | gaattgcggcatttttcaat |
| -7.221kb | gtcctcactttgggagtgga | ggggtcagcagatgtgaact |
| -6.720kb | acacctgctcagctggaatc | gtctggaggctgggaagaat |
| -5.888kb | gtgaccttctggcttgaacc | accttttctcccactttgtcc |
| -5.169kb | gttttcctcccagggttctc | gtggccccttcactacttga |
| -4.430kb | gccatgacaatgtcctaccc | acttggtgtcggaatctcca |
| -3.552kb | aaaagcaggtgaggacgtgt | gactgtgtctggctgtggaa |
| -2.004kb | GGGTCTAAGGCCAGAAGTGA | TGTGGTGGAAAACCTGCTG |
| -1.390kb | ACCTGGGACCAGTGAGTGAG | AGAGTCTGCTGTTGGCATCC |
| -0.796kb | CTCCAGGGGCTCCAAAGTA | AGGCAGGGACAGCTGAGAG |
| -0.588kb | AGGAGACCGATCCTGAGACC | GCCATACCTGCCTGTCTTTG |
| -0.124kb | GAGCCCAGGGGACACACCT | CTCGCCTTACAAGGAGCAGT |
| +0.180kb | CTGGCTGAGGCAGAGCAC | ACCGGCTTCGGAAGGAAT |
| +0.526kb | ACTGCAGACACAGGCTGGA | GTCCAAGATGGCTCCACAC |
| +0.980kb | GTGGCTGGTCTGGTACAGGT | AACCTCACGAGAGACGTTGC |
| +2.148kb | GCGGTGATGATTAACCCAAC | AAACGGACCTTCCCTGTTTT |
| +2.846kb | CTCTGCTCTGAACGGGAAGT | CCCATCAAATCAGTGCTTCA |
| +3.303kb | GGAAGGAGGACAGGAAGGAC | AGGCTTCTACTCCAGGCACA |
| +4.996kb | CTTACCGGACAGTGCTGGAT | gagtaggagctccggatgtg |
| +6.147kb | ATGGTTAGACCTGGCCCTCT | gcaggcagctctctcctcta |
| +7.722kb | TGTGGTCAGCTCAGGTCAAG | GTAGCTCCCTTCAGCACAGG |
